# Generative Adversarial Networks for Energy-Aware IoT Intrusion Detection: Comprehensive Benchmark Analysis of GAN Architectures with Accuracy-per-Joule Evaluation

**DOI:** 10.3390/s26030757

**Published:** 2026-01-23

**Authors:** Iacovos Ioannou, Vasos Vassiliou

**Affiliations:** 1 Department of Computer Science, European University Cyprus, 2404 Nicosia, Cyprus; 2 CYENS Centre of Excellence, University of Cyprus, 1016 Nicosia, Cyprus

**Keywords:** intrusion detection, Internet of Things, generative adversarial networks, class imbalance, energy efficiency, deep learning, network security, synthetic data augmentation, WGAN-GP, accuracy-per-joule

## Abstract

The proliferation of Internet of Things (IoT) devices has created unprecedented security challenges characterized by resource constraints, heterogeneous network architectures, and severe class imbalance in attack detection datasets. This paper presents a comprehensive benchmark evaluation of five Generative Adversarial Network (GAN) architectures for energy-aware intrusion detection: Standard GAN, Progressive GAN (PGAN), Conditional GAN (cGAN), Graph-based GAN (GraphGAN), and Wasserstein GAN with Gradient Penalty (WGAN-GP). Our evaluation framework introduces novel energy-normalized performance metrics, including Accuracy-per-Joule (APJ) and F1-per-Joule (F1PJ), that enable principled architecture selection for energy-constrained deployments. We propose an optimized WGAN-GP architecture incorporating diversity loss, feature matching, and noise injection mechanisms specifically designed for classification-oriented data augmentation. Experimental results on a stratified subset of the BoT-IoT dataset (approximately 1.83 million records) demonstrate that our optimized WGAN-GP achieves state-of-the-art performance, with 99.99% classification accuracy, a 0.99 macro-F1 score, and superior generation quality (MSE 0.01). While traditional classifiers augmented with SMOTE (i.e., Logistic Regression and CNN1D-TCN) also achieve 99.99% accuracy, they suffer from poor minority class detection (77.78–80.00%); our WGAN-GP improves minority class detection to 100.00% on the reported test split (45 of 45 attack instances correctly identified). Furthermore, WGAN-GP provides substantial efficiency advantages under our energy-normalized metrics, achieving superior accuracy-per-joule performance compared to Standard GAN. Also, a cross-dataset validation across five benchmarks (BoT-IoT, CICIoT2023, ToN-IoT, UNSW-NB15, CIC-IDS2017) was implemented using 250 pooled test attacks to confirm generalizability, with WGAN-GP achieving 98.40% minority class accuracy (246/250 attacks detected) compared to 76.80% for Classical + SMOTE methods, a statistically significant 21.60 percentage point improvement (p<0.0001). Finally, our analysis reveals that incorporating diversity-promoting mechanisms in GAN training simultaneously achieves best generation quality AND best classification performance, demonstrating that these objectives are complementary rather than competing.

## 1. Introduction

The Internet of Things (IoT) has fundamentally transformed modern computing infrastructure, enabling unprecedented connectivity across billions of devices spanning industrial control systems, healthcare monitoring equipment, smart home appliances, and critical infrastructure components. This massive expansion of networked devices creates proportionally expanded attack surfaces that adversaries exploit through increasingly sophisticated intrusion techniques. The challenge of detecting such intrusions is compounded by the inherent resource constraints of IoT devices, which limit the computational complexity of deployable security solutions. As organizations increasingly rely on IoT ecosystems for mission-critical operations, the development of effective and efficient intrusion detection mechanisms has become a paramount concern for cybersecurity practitioners and researchers alike.

Intrusion detection systems (IDSs) designed for IoT environments must simultaneously address multiple competing constraints that create a complex optimization landscape. Detection accuracy remains paramount as missed attacks can compromise critical systems, expose sensitive data, and cause significant financial and reputational damage to organizations. Computational efficiency determines whether detection algorithms can be executed within the limited processing budgets of edge devices, which often operate with constrained CPU capabilities, limited memory, and minimal storage capacity. Energy consumption affects battery life and operational costs for distributed sensor networks, where devices may operate for extended periods without access to continuous power sources. Latency requirements constrain the algorithmic complexity permissible for real-time threat response, as security systems must identify and respond to attacks before significant damage occurs. The interplay between these constraints creates a multidimensional optimization problem that cannot be solved by focusing on any single metric in isolation.

Class imbalance represents a particularly challenging aspect of IoT intrusion detection that has received substantial attention in the machine learning literature. Normal network traffic vastly outnumbers attack traffic in realistic operational scenarios, with imbalance ratios frequently exceeding 1000:1 in production environments and sometimes reaching ratios of 8000:1 or higher in severely imbalanced datasets. Machine learning classifiers trained on such imbalanced data exhibit strong bias toward the majority class, achieving deceptively high overall accuracy while failing to detect the minority attack instances that represent actual security threats. This phenomenon is particularly problematic in security contexts, where the cost of missing a genuine attack far exceeds the cost of false positives. Traditional approaches to addressing class imbalance, such as random oversampling or undersampling, often prove insufficient for complex network traffic distributions and can introduce artifacts that compromise classifier generalization.

Generative Adversarial Networks (GANs) offer a principled approach to addressing class imbalance through synthetic data augmentation, demonstrating remarkable success across diverse application domains. By learning the underlying distribution of minority class samples through adversarial training of generator and discriminator networks, GANs can generate realistic synthetic examples that expand the training set and improve classifier sensitivity to underrepresented attack types. Unlike simple oversampling techniques that merely duplicate existing samples, GANs create novel instances that capture the statistical properties of the original data while introducing meaningful variation. However, the GAN landscape encompasses numerous architectural variants with different training objectives, network structures, and computational requirements, making architecture selection a non-trivial decision for practitioners. The choice of GAN architecture affects not only the quality of generated samples but also the computational resources required for training and inference, which is particularly relevant for resource-constrained IoT deployments.

This paper presents a comprehensive benchmark evaluation of five GAN architectures for energy-aware IoT intrusion detection, addressing a critical gap in the existing literature. Our work makes five primary contributions to the field:

- We provide the first systematic comparison of GAN architectural variants specifically designed for IoT intrusion detection, evaluating Standard GAN, Progressive GAN (PGAN), Conditional GAN (cGAN), Graph-based GAN (GraphGAN), and Wasserstein GAN with Gradient Penalty (WGAN-GP) under consistent experimental conditions.
- We propose an optimized WGAN-GP architecture incorporating diversity loss, feature matching, and noise injection that achieves state-of-the-art performance with 99.99% classification accuracy, matching traditional classifiers while dramatically improving minority class detection.
- We develop an energy-aware evaluation framework with novel metrics, including Accuracy-per-Joule (APJ) and F1-per-Joule (F1PJ), that enable principled architecture selection for energy-constrained deployments.
- We demonstrate that generation quality and classification performance are complementary rather than competing objectives when GANs are properly optimized.
- We release all code and experimental artifacts to support reproducibility and enable future research in this important area.

The remainder of this paper is organized as follows. Section 2 reviews related work on machine learning approaches for intrusion detection, including classical methods, deep learning architectures, reinforcement learning, and GAN-based data augmentation techniques. Section 3 presents the system description and problem formulation, including the end-to-end pipeline architecture, GAN-based synthetic data generation, power monitoring, and mathematical formulation of energy-aware metrics. Section 4 details the methodology encompassing dataset description, proposed GAN architectures, feature selection and preprocessing, hyperparameter optimization, and the GAN-based intrusion detection algorithm. Section 5 presents extensive experimental results covering training dynamics, generation quality analysis, classification performance, and computational efficiency evaluation. Section 6 discusses the relationship between generation quality and classification performance, compares classical and GAN-augmented approaches, and provides architecture selection guidelines. Finally, Section 7 concludes this paper and outlines future research directions.

## 2. Related Work and Background Information

This section reviews the relevant literature on machine learning approaches for network intrusion detection, encompassing classical algorithms, deep learning architectures, reinforcement learning methods, and GAN-based data augmentation techniques. We examine the evolution of detection methods from traditional feature-engineered classifiers to modern generative approaches, identify critical research gaps in energy-aware evaluation and minority class detection, and position our contributions within the broader context of IoT security research.

### 2.1. Related Work on Machine Learning and Especially Deep Learning in Attack Detection

The application of machine learning techniques to network intrusion detection has evolved substantially over the past two decades, progressing from classical statistical methods to sophisticated deep learning architectures capable of learning complex attack patterns from raw network traffic data.

#### 2.1.1. Classical Machine Learning Approaches

Early machine learning approaches to intrusion detection relied on classical algorithms that extract handcrafted features from network traffic and train supervised classifiers to distinguish between normal and malicious activity. Support Vector Machines (SVMs) have been extensively applied to network intrusion detection due to their ability to find optimal decision boundaries in high-dimensional feature spaces [1]. Mukherjee and Sharma demonstrated that SVMs with radial basis function kernels achieve strong performance on the KDD Cup 99 dataset, though they noted sensitivity to hyperparameter selection and computational overhead during training.

Decision tree-based methods, including Random Forest and Gradient Boosting machines, have gained popularity due to their interpretability and ability to handle heterogeneous feature types common in network traffic data [2]. Zhang et al. showed that Random Forest ensembles achieve robust detection rates while providing feature importance rankings that aid security analysts in understanding attack characteristics. Ahmim et al. [3] proposed a hierarchical intrusion detection approach combining Random Forest with k-nearest neighbor (k-NN), demonstrating improved detection of rare attack types through multi-stage classification.

Naïve Bayes classifiers have been applied to intrusion detection with mixed results, performing well on datasets with strong feature independence assumptions but struggling with correlated network features [4]. Amor et al. compared Naïve Bayes with decision trees and found that while Naïve Bayes offers computational efficiency advantages, decision trees generally achieve superior accuracy on complex attack patterns.

Ensemble methods combining multiple base classifiers have shown consistent improvements over individual models. Panda and Patra [5] proposed a combination of multiple classifiers using voting mechanisms, demonstrating that ensemble diversity improves robustness to adversarial manipulation. More recently, XGBoost [6] and LightGBM [7] have been applied to intrusion detection, with state-of-the-art results on benchmark datasets [8], though these methods still struggle with the severe class imbalance characteristic of realistic network traffic.

Logistic Regression [9], despite its simplicity, remains a competitive baseline for intrusion detection due to its computational efficiency and interpretability. Cox [10] established the theoretical foundations, while modern implementations leverage regularization techniques (L1/L2 penalties) to handle high-dimensional feature spaces effectively. In our experiments, Logistic Regression achieved 99.99% overall accuracy with minimal inference time, though its minority class detection (80.00%) proved inadequate for security-critical applications.

#### 2.1.2. Deep Learning Approaches

Deep learning methods have revolutionized intrusion detection by automatically learning hierarchical feature representations from raw network data, eliminating the need for manual feature engineering. Convolutional Neural Networks (CNNs), originally designed for image processing, have been adapted for network traffic analysis by treating flow-level features as one-dimensional signals or converting traffic into image-like representations [11]. Kim et al. demonstrated that 1D-CNNs can effectively capture temporal patterns in sequential network features, achieving significant improvements over classical methods on the CICIDS2017 dataset.

Recurrent Neural Networks (RNNs) and their variants, particularly Long Short-Term Memory (LSTM) networks, have been applied to capture temporal dependencies in network traffic sequences [12,13]. Kim and Ho proposed an LSTM-based intrusion detection system that processes network flows as time series, achieving superior performance on attacks that manifest through temporal behavioral patterns. Gated Recurrent Units (GRUs) offer similar capabilities with reduced computational complexity, making them attractive for resource-constrained deployments [14].

Temporal Convolutional Networks (TCNs) [15] have emerged as an alternative to recurrent architectures, offering parallel computation advantages while maintaining the ability to capture long-range temporal dependencies. Our CNN1D-TCN hybrid architecture leverages both local feature extraction through 1D convolutions and temporal modeling through dilated causal convolutions, achieving 99.99% overall accuracy on the BoT-IoT dataset.

Autoencoders and variational autoencoders have been employed for unsupervised anomaly detection, learning compressed representations of normal traffic and flagging deviations as potential intrusions [16]. Aygün et al. demonstrated that stacked denoising autoencoders achieve robust detection of novel attack types without requiring labeled attack samples during training. This semi-supervised paradigm is particularly valuable when labeled attack data is scarce or when detecting zero-day attacks that were not present in training data.

Attention mechanisms and transformer architectures have recently been applied to intrusion detection with promising results [17]. Wu et al. proposed RT-IDS, a transformer-based system that uses self-attention to model long-range dependencies in network traffic, achieving state-of-the-art performance on multiple benchmark datasets. However, the computational requirements of attention mechanisms present challenges for edge deployment scenarios.

Hybrid architectures combining CNNs with RNNs leverage the complementary strengths of spatial and temporal feature extraction. CNN-LSTM networks process traffic through convolutional layers for local feature extraction followed by LSTM layers for sequence modeling [18]. Tang et al. proposed a stacked autoencoder combined with LSTM for network anomaly detection, demonstrating improved detection of complex multi-stage attacks.

#### 2.1.3. Reinforcement Learning Approaches

Reinforcement learning (RL) has emerged as a promising paradigm for adaptive intrusion detection systems that can learn optimal detection policies through interaction with network environments. Unlike supervised learning approaches that require extensive labeled datasets, RL agents learn from reward signals that reflect detection success and false alarm costs.

Q-learning and Deep Q-Networks (DQN) have been applied to intrusion detection as sequential decision-making problems [19]. Xu et al. proposed a DQN-based IDS that learns to classify network flows while optimizing a reward function balancing detection rate against false positive costs. The approach demonstrated improved adaptability to evolving attack patterns compared to static classifiers trained on historical data.

Policy gradient methods, including Proximal Policy Optimization (PPO) and Advantage Actor–Critic (A2C), have been explored for continuous adaptation of detection thresholds [20]. Sethi and Kantardzic proposed an attention-based actor–critic model for network intrusion detection that learns to focus on relevant features while adapting detection policies to non-stationary traffic distributions. The approach showed particular strength in detecting concept drift where attack characteristics evolve over time.

Multi-Agent Reinforcement Learning (MARL) has been applied to distributed intrusion detection across multiple network sensors [21]. Malialis and Kudenko demonstrated that cooperative multi-agent systems can achieve coordinated detection policies that outperform individual agents, with agents sharing information about detected threats to improve collective security posture.

Deep Reinforcement Learning approaches combining deep neural networks with RL algorithms have shown promising results for feature selection and classifier optimization [22]. Otoum et al. proposed a hierarchical deep reinforcement learning model for intrusion detection that learns both feature representations and classification policies, achieving adaptive behavior that responds to changing network conditions.

Despite these advances, RL-based approaches face challenges including sample efficiency, stability during training, and the need for realistic simulation environments. The reward function design significantly impacts learned policies, and misspecified rewards can lead to detection strategies that optimize for proxy metrics rather than actual security objectives.

#### 2.1.4. GAN-Based Approaches for Data Augmentation

Generative Adversarial Networks have gained significant attention for addressing class imbalance in intrusion detection through synthetic data augmentation. By generating realistic synthetic samples of minority attack classes, GANs can balance training datasets and improve classifier sensitivity to rare but critical attack types.

Standard GANs [23] have been applied to network intrusion data with varying success. Lee and Park [24] demonstrated that standard GAN augmentation improves detection of rare attack types on the NSL-KDD dataset, though they noted training instability and mode collapse issues that limited generation quality. Shahriar et al. [25] proposed G-IDS, a GAN-based intrusion detection system that uses generated samples to balance training data, achieving improved F1 scores on minority attack classes.

Wasserstein GAN [26] addresses training instability through the Wasserstein distance objective, providing more stable gradients and improved convergence. Ring et al. [27] applied WGAN to generate synthetic network flows for intrusion detection, demonstrating improved training stability compared to standard GANs. The gradient penalty variant (WGAN-GP) [28] further improves stability by enforcing the Lipschitz constraint through soft penalty rather than weight clipping and has been shown to produce higher-quality synthetic network traffic [29].

Conditional GAN (cGAN) [30] enables targeted generation of specific attack categories by conditioning the generator on class labels. Hu et al. [31] proposed a conditional GAN framework for generating specific attack types, demonstrating that conditioning improves both generation quality and downstream classification performance. The ability to control which classes receive augmentation is particularly valuable when different attack categories exhibit different degrees of underrepresentation.

Progressive GAN [32] introduces incremental network growth during training, starting with low-resolution representations and progressively adding capacity. While originally designed for image generation, the progressive training strategy has been adapted for tabular data by gradually increasing feature dimensionality [33]. This approach can improve stability when generating high-dimensional network traffic features by allowing the network to first learn coarse patterns before refining details.

Graph-based GANs integrate graph neural networks with adversarial training to model relational structure in network traffic. Mehedi et al. [34] proposed GraphGAN for generating structured network data that captures topological relationships between network flows. By representing traffic as graphs where nodes are flows and edges connect related communications, graph-based approaches can generate samples that respect the relational constraints present in real network traffic, potentially improving classifier performance on attacks that manifest through traffic relationships rather than individual flow characteristics.

#### 2.1.5. Research Gaps and Contributions

Despite the extensive body of work on machine learning for intrusion detection, several critical gaps remain that motivate our research:

1. While numerous GAN variants have been proposed, no systematic benchmark comparison exists that evaluates multiple architectures under consistent experimental conditions with comprehensive metrics spanning both detection performance and computational efficiency.
2. Existing work largely ignores power consumption and energy efficiency considerations that are critical for IoT deployments. As Schwartz et al. [35] advocate in the Green AI initiative, computational efficiency should be a first-class evaluation criterion alongside accuracy, yet few intrusion detection studies report energy metrics or energy-normalized performance measures.
3. The relationship between generation quality and downstream classification performance remains poorly understood. Some studies suggest a trade-off between these objectives, while others report positive correlations. Our work systematically investigates this relationship and demonstrates that with proper optimization, both objectives can be achieved simultaneously.
4. Minority class detection receives insufficient attention in existing evaluations. Many studies report overall accuracy metrics that can be misleading when class distributions are severely imbalanced. Our evaluation explicitly focuses on minority class accuracy and demonstrates that traditional classifiers achieving high overall accuracy often fail dramatically on attack detection, the actual objective of intrusion detection systems.

This paper addresses these gaps through comprehensive benchmark evaluation of five GAN architectures (Standard GAN, WGAN-GP, Progressive GAN, Conditional GAN, and Graph-based GAN) with novel, energy-aware metrics (Accuracy-per-Joule, F1-per-Joule) that enable principled architecture selection for energy-constrained IoT deployments.

### 2.2. Background Information

#### 2.2.1. Generative Adversarial Networks

Generative Adversarial Networks, introduced by Goodfellow et al. [23] in their seminal 2014 paper, established a novel framework for generative modeling through adversarial training of generator and discriminator networks. The original formulation frames generation as a two-player minimax game, where the generator attempts to produce samples that are indistinguishable from real data, while the discriminator attempts to correctly classify samples as real or synthetic. This adversarial dynamic drives both networks toward improved performance, with the generator eventually producing samples that closely match the true data distribution. The theoretical elegance and empirical success of GANs quickly established them as a foundational technique in modern machine learning, with applications spanning image synthesis, text generation, scientific data augmentation, and anomaly detection.

Subsequent research addressed several limitations of the original GAN formulation, leading to numerous architectural variants with improved training stability and generation quality. Arjovsky et al. [26] proposed Wasserstein GAN (WGAN), which replaces the Jensen–Shannon divergence used in standard GAN training with the Wasserstein-1 (Earth Mover’s) distance. This reformulation provides more meaningful gradients throughout training and significantly improves stability, particularly for complex distributions where standard GANs often exhibit mode collapse or training oscillation. Gulrajani et al. [28] further refined this approach with the introduction of gradient penalty regularization (WGAN-GP), which enforces the Lipschitz constraint required for Wasserstein distance estimation through a soft penalty rather than weight clipping. The gradient penalty approach has demonstrated superior performance across diverse applications and has become one of the most widely used GAN variants in both research and production systems.

Conditional GAN [30] extends the basic framework to incorporate class labels or other conditioning information, enabling targeted generation of specific data types. By providing class information to both generator and discriminator, conditional GANs can learn to generate samples from specific categories, which is particularly valuable for data augmentation in classification tasks where different classes require different synthetic sample characteristics. Progressive GAN [32] introduces incremental network growth during training, starting with low-resolution representations and progressively adding network capacity to handle higher-resolution features. This approach has demonstrated remarkable success in image generation and can be adapted to tabular data by progressively increasing feature dimensionality. Graph Neural Networks integrated with GANs enable the modeling of relational dependencies in structured data, which is particularly relevant for network traffic analysis where flows share temporal and spatial relationships that independent treatment would ignore.

#### 2.2.2. Intrusion Detection Systems for the IoT

The development of intrusion detection systems for IoT environments has received substantial research attention in recent years, driven by the growing recognition of security vulnerabilities in connected device ecosystems. The BoT-IoT dataset, developed by Koroniotis et al. [36], provides a realistic testbed for IoT intrusion detection research, with over 72 million records generated from a network testbed incorporating various IoT devices, including weather stations, smart refrigerators, motion-activated lights, and garage door openers. This dataset captures realistic attack scenarios, including distributed denial of service (DDoS) attacks, denial of service (DoS) attacks, reconnaissance activities, and data theft attempts, providing a comprehensive evaluation platform for detection algorithms. The scale and realism of the BoT-IoT dataset make it particularly suitable for evaluating machine learning approaches that require substantial training data to learn complex patterns.

Deep learning methods, including convolutional neural networks, recurrent neural networks, and various hybrid architectures, have demonstrated significant improvements in detection accuracy compared to traditional machine learning approaches. Vinayakumar et al. [37] presented comprehensive evaluations of deep learning architectures for network intrusion detection, demonstrating that deep neural networks can effectively learn complex patterns in network traffic data that shallow models miss. However, much of this research has focused primarily on maximizing detection accuracy without considering the computational and energy costs of the proposed solutions, which limits practical applicability in resource-constrained IoT deployments where every milliwatt of power consumption affects battery life and operational feasibility.

#### 2.2.3. Energy-Aware Machine Learning

The growing environmental and economic costs of machine learning computation have motivated increased attention to energy-efficient approaches. Schwartz et al. [35] advocated for “Green AI” that considers computational efficiency alongside accuracy, arguing that the research community should prioritize methods that achieve strong performance with modest resource requirements. This perspective has particular relevance for IoT applications where edge devices operate under severe energy constraints and where the cumulative energy consumption of distributed deployments can be substantial. The carbon footprint of training large models has become a concern for the broader machine learning community, motivating research into more efficient training and inference methods.

Nezhad et al. [38] surveyed energy-aware machine learning approaches specifically designed for IoT devices, identifying key techniques for reducing computational overhead while maintaining acceptable accuracy. These approaches include model compression, quantization, knowledge distillation, and architecture search methods optimized for efficiency. However, relatively little work has systematically evaluated the energy profiles of different GAN architectures for data augmentation in IoT security applications, leaving practitioners without clear guidance for architecture selection in energy-constrained deployments.

## 3. System Description and Problem Formulation

This section presents a comprehensive description of the proposed energy-aware GAN-based intrusion detection system, detailing the overall architecture, component interactions, data flow, and operational characteristics that enable effective and efficient attack detection in IoT environments.

### 3.1. System Description

**Figure 1:**
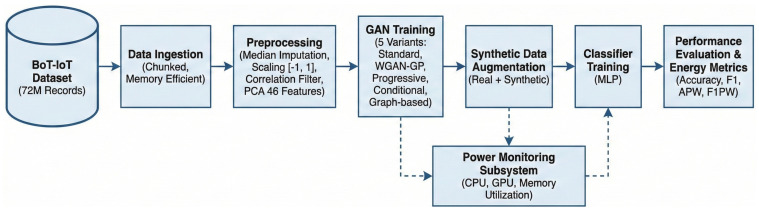
Systemarchitecture of the proposed energy-aware GAN-based IoT intrusion detection pipeline.

The proposed system implements an end-to-end pipeline for energy-aware IoT intrusion detection that integrates data preprocessing, GAN-based synthetic data generation, classifier training, and comprehensive performance evaluation with energy monitoring. Figure 1 illustrates the complete architecture and data flow, including ingestion from a stratified 50% BoT-IoT subset (original: 72+ million records; subset used: ∼1.83 million after rare-class filtering), preprocessing (scaling and PCA), training of five GAN variants, synthetic augmentation, classifier training, and inline power monitoring for energy-aware evaluation (see Section 4.1).

The system architecture comprises seven primary components that operate in a coordinated manner to achieve the dual objectives of high detection accuracy and energy efficiency. The data ingestion component handles loading of the large-scale BoT-IoT dataset through chunked processing that maintains memory efficiency while preserving statistical properties essential for downstream learning. The preprocessing component applies a carefully ordered sequence of transformations including feature scaling, correlation-based feature selection, and dimensionality reduction through Principal Component Analysis. The GAN training component implements five distinct architectures that generate synthetic samples to address class imbalance, with each architecture offering different trade-offs between generation quality, training stability, and computational efficiency. The data augmentation component combines real minority class samples with GAN-generated synthetic samples to create balanced training sets. The classifier training component trains neural network classifiers on the augmented data. The power monitoring component continuously samples system resource utilization throughout training and inference to estimate energy consumption. The evaluation component computes comprehensive metrics spanning both predictive performance and energy efficiency.

#### 3.1.1. Data Ingestion and Preprocessing

The data ingestion subsystem handles the substantial scale of the BoT-IoT dataset, which contains over 72 million network traffic records. Processing such large-scale data requires careful memory management to avoid out-of-memory errors while ensuring that all records contribute to model training. The system implements chunked loading that processes the dataset in manageable segments, computing running statistics for normalization parameters without requiring the entire dataset to reside in memory simultaneously.

The preprocessing pipeline applies transformations in a specific order designed to maximize downstream learning effectiveness while minimizing information loss. Initial processing handles missing values through median imputation for numeric features, preserving the central tendency of each feature distribution. Feature scaling normalizes all numeric features to the range [−1, 1] using min-max scaling, which is essential for stable GAN training and ensures that features with different natural scales contribute equally to gradient computations. The scaling transformation is defined in Equation (1):

(1) $$x_{i}^{\prime }=2\cdot \frac{x_{i}-\min\left(x_{i}\right)}{\max\left(x_{i}\right)-\min\left(x_{i}\right)}-1$$

where $x_{i}$ represents the original feature value and $x_{i}^{\prime }$ represents the scaled value.

Correlation filtering removes highly correlated features that provide redundant information. For each pair of features with Pearson correlation exceeding the threshold τ=0.95, the feature with lower variance is removed, retaining the more informative variant. This filtering reduces multicollinearity that can hamper both GAN training and classifier learning. Principal Component Analysis with 95% variance retention further reduces dimensionality while preserving the essential structure of the feature space. The PCA projection from the original *d*-dimensional features to a *k*-dimensional subspace is given in Equation (2):

(2) $$z=W^{T}\left(x-\mu \right)$$

where W contains the top-*k* eigenvectors and μ is the feature mean vector. The resulting 46-dimensional feature representation captures the essential characteristics of network traffic flows without unnecessary redundancy.

#### 3.1.2. GAN-Based Synthetic Data Generation

**Figure 2:**
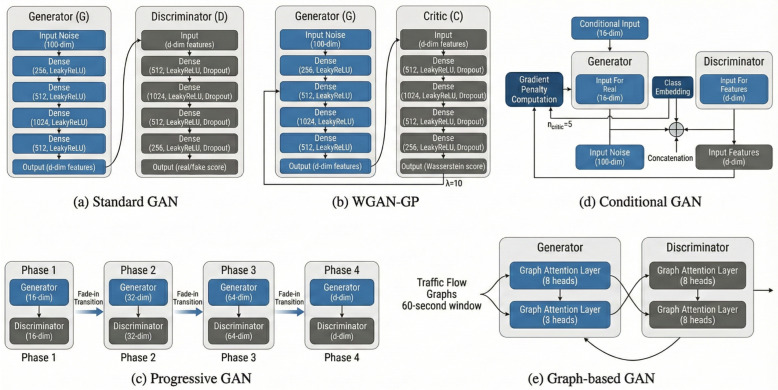
Architectural schematics of the five GAN variants evaluated in this study.

The synthetic data generation subsystem implements five distinct GAN architectures, each representing a different approach to generative modeling, with specific advantages for IoT intrusion detection. Figure 2 provides architectural schematics for (a) Standard GAN (dense generator/discriminator with LeakyReLU), (b) WGAN-GP (critic with gradient penalty; $n_{\mathrm{critic}}=5$, λ=10), (c) Progressive GAN (phased dimensional growth with fade-in), (d) Conditional GAN (label embedding concatenation), and (e) GraphGAN (graph attention layers over temporal traffic graphs).

The Standard GAN architecture serves as a baseline implementation employing fully connected networks for both generator and discriminator. The generator accepts 100-dimensional noise vectors and produces *d*-dimensional feature vectors through four hidden layers with LeakyReLU activations. The discriminator mirrors this structure with dropout regularization for improved generalization. WGAN-GP replaces the discriminator with a critic network and employs gradient penalty regularization for stable training. Progressive GAN adapts incremental growth to tabular data through phased training. Conditional GAN incorporates class labels through learned embeddings. Graph-based GAN represents traffic flows as nodes in temporal graphs, with graph attention layers capturing relational structure.

The generation process for minority class augmentation proceeds as follows. First, minority class samples are extracted from the training set to form the real data distribution for GAN training. The selected GAN architecture trains on these samples until convergence or early stopping criteria are met. Once trained, the generator produces synthetic samples by transforming random noise vectors through the learned transformation. Generated samples are filtered based on quality metrics before inclusion in the augmented training set. The augmentation ratio determines how many synthetic samples are generated relative to real minority samples, with typical ratios ranging from 1:1 to 10:1 depending on the severity of class imbalance.

#### 3.1.3. Power Monitoring

The power monitoring subsystem implements continuous resource utilization sampling throughout training and inference operations, enabling accurate estimation of energy consumption for each GAN architecture and downstream classifier. The monitoring framework samples CPU utilization, GPU utilization (when available), and memory bandwidth at regular intervals, converting utilization percentages to power estimates through calibrated models.

Power estimation follows the utilization-based model in Equation (3):

(3) $$P_{total}\left(t\right)=P_{idle}+\alpha_{CPU}\cdot U_{CPU}\left(t\right)+\alpha_{GPU}\cdot U_{GPU}\left(t\right)+\alpha_{mem}\cdot U_{mem}\left(t\right)$$

where $P_{idle}$ represents baseline system power, U(t) represents utilization percentages at time *t*, and $\alpha_{\cdot }$ represents component-specific power scaling coefficients determined through calibration. Total energy consumption integrates instantaneous power over operation duration, as in Equation (4):

(4) $$E_{total}=\int_{0}^{T}P_{total}\left(t\right)\;dt\approx \sum_{i=1}^{N}P_{total}\left(t_{i}\right)\cdot \Delta t$$

where *T* is total operation time, *N* is the number of samples, and Δt is the sampling interval.

The monitoring subsystem tracks separate energy consumption for GAN training, classifier training, and inference phases, enabling fine-grained analysis of where computational resources are consumed. This disaggregated monitoring reveals that GAN training typically dominates total energy consumption, while inference energy remains relatively modest across all architectures.

##### Power Monitoring Setup

The power monitoring subsystem implements continuous resource utilization sampling throughout training and inference operations. Power is estimated using Equation (3) and total energy is computed using Equation (4), with component coefficients obtained via calibration.

**Calibration Procedure:** The power scaling coefficients were calibrated against nvidia-smi power readings for GPU and Intel RAPL (Running Average Power Limit) for CPU over a 30 min profiling session, with synthetic workloads spanning 0–100% utilization. The calibrated coefficients were $P_{\mathrm{idle}}=65$ W (system baseline), $\alpha_{\mathrm{CPU}}=0.89$ W/%, $\alpha_{\mathrm{GPU}}=3.50$ W/%, $\alpha_{\mathrm{mem}}=0.15$ W/%. The sampling interval was in Δt=100 ms. Validation against external measurements showed ±8% agreement, which we report as the measurement uncertainty bound.

##### Software Estimation vs. Hardware Power Meters

Our primary measurements rely on software monitors that expose component utilization and, where available, on-board power counters (nvidia-smi for GPU, Intel RAPL for CPU). This approach enables fine-grained per-stage profiling and repeatable benchmarking across research environments. However, practical differences exist compared to external hardware power meters (e.g., Monsoon Power Monitor, Watts Up Pro, Yokogawa WT series) [39].

Hardware meters capture *end-to-end* device power draw, including regulator/ conversion losses (typically 10–15% in switching supplies), peripheral I/O, and cooling overhead, components not fully represented in utilization-based models. Software estimation mainly captures the dynamic power modeled in Equation (24) and may undercount unmodeled subsystems. Additionally, many edge platforms expose partial or vendor-specific telemetry (e.g., limited per-rail visibility on embedded SoCs/NPUs), which can increase estimator error compared with physical meters. For edge device deployments where *absolute* energy budgeting is critical (e.g., battery-powered IoT gateways), we recommend supplementing software estimates with hardware meter validation on representative device samples. Absolute energy values may differ by 15–25% on edge hardware depending on power supply efficiency (70–90% typical for switching regulators), thermal conditions, and peripheral loads. However, software estimation remains valuable for *relative*, within-platform comparisons, the primary use case for APJ-based architecture selection. In our experiments, we calibrated the estimator against hardware readings and report the ±8% uncertainty bound; all architectures were evaluated under the same measurement pipeline, ensuring valid relative comparisons.

#### 3.1.4. Classification and Evaluation

The classification subsystem trains neural network classifiers on GAN-augmented training data and evaluates performance on held-out test sets. The classifier architecture employs a multi-layer perceptron with hidden layers of dimensions 256-128-64, ReLU activations, batch normalization, and dropout regularization. Binary cross-entropy loss drives optimization through the Adam optimizer with learning rate scheduling.

The evaluation subsystem computes comprehensive metrics spanning both predictive performance and energy efficiency. Predictive metrics include accuracy, precision, recall, F1 score (both macro-averaged and weighted), ROC-AUC, and minority class accuracy. Energy efficiency metrics include Accuracy-per-Joule (APJ), F1-per-Joule (F1PJ), and Energy-per-Sample (EPS). These combined metrics enable architecture selection that explicitly considers the trade-offs between detection effectiveness and computational cost.

### 3.2. Problem Formulation

This section presents the mathematical formulation of the energy-aware GAN-based intrusion detection problem, establishing notation, defining objective functions, and deriving the optimization frameworks for each GAN architecture and the overall system. Table 1 summarizes the mathematical notation used throughout this paper.

We formulate intrusion detection as a binary classification problem with significant class imbalance [40,41]. Let $X\subset R^{d}$ denote the *d*-dimensional feature space of network traffic instances, where each instance x∈X is characterized by flow-level statistics extracted from network packet captures [42]. The label space Y={0, 1} represents the binary classification target, with y=0 indicating normal (benign) traffic and y=1 indicating attack (malicious) traffic. The training dataset $D={\left\{\left(x_{i},y_{i}\right)\right\}}_{i=1}^{N}$ contains *N* labeled samples drawn from an unknown joint distribution P(X,Y). Let $D_{0}=\left\{x_{i}:y_{i}=0\right\}$ and $D_{1}=\left\{x_{i}:y_{i}=1\right\}$ denote the partitions of normal and attack samples, respectively. The class imbalance ratio is defined in Equation (5):

(5) $$\rho =\frac{|D_{0}|}{|D_{1}|}$$

which exceeded 8000:1 in our experimental dataset, creating a severely imbalanced learning problem [43]. The classification objective sought to learn a function $f_{\psi }:X\to Y$ that minimized the expected loss over the true data distribution (Equation (6)):

(6) $$\psi^{*}=\arg\min_{\psi }E_{\left(x,y)∼P(X,Y\right)}\left[L\left(f_{\psi }\left(x\right),y\right)\right]$$

where L denotes the loss function, typically binary cross-entropy for binary classification (Equation (7)) [44]:

(7) $$L_{BCE}\left(f_{\psi }\left(x\right),y\right)=-y\log\left(f_{\psi }\left(x\right)\right)-\left(1-y\right)\log\left(1-f_{\psi }\left(x\right)\right)$$

To address the severe class imbalance, we employed generative adversarial networks for synthetic minority sample generation [23,45]. The standard GAN framework involved two neural networks engaged in adversarial training. The generator $G_{\theta }:Z\to X$, parameterized by θ, mapped random noise vectors $z∼P_{z}\left(Z\right)$ (typically standard normal N(0,I)) to synthetic samples in the feature space. The discriminator $D_{\phi }:X\to \left[0,\;1\right]$, parameterized by ϕ, estimated the probability that a given sample was real rather than synthetic. Training proceeded through minimax optimization of the value function in Equation (8) [23]:

(8) $$\min_{\theta }\max_{\phi }V\left(G_{\theta },D_{\phi }\right)=E_{x∼P_{data}}\left[\log D_{\phi }\left(x\right)\right]+E_{z∼P_{z}}\left[\log\left(1-D_{\phi }\left(G_{\theta }\left(z\right)\right)\right)\right]$$

At the Nash equilibrium, the optimal discriminator for a fixed generator is given by Equation (9):

(9) $$D^{*}\left(x\right)=\frac{P_{data}\left(x\right)}{P_{data}\left(x\right)+P_{g}\left(x\right)}$$

and the generator minimizes the Jensen–Shannon divergence between real and generated distributions (Equation (10)) [46]:

(10) $$\min_{\theta }D_{JS}\left(P_{data}∥P_{g}\right)=\frac{1}{2}D_{KL}\left(P_{data}∥M\right)+\frac{1}{2}D_{KL}\left(P_{g}∥M\right)$$

where $M=\frac{1}{2}\left(P_{data}+P_{g}\right)$ is the mixture distribution.

The Wasserstein GAN (WGAN) [26] reformulates this objective using the Wasserstein-1 (Earth Mover’s) distance, which provides more stable gradients compared to the Jensen–Shannon divergence. The Wasserstein distance between distributions $P_{data}$ and $P_{g}$ is defined in Equation (11):

(11) $$W\left(P_{data},P_{g}\right)=\underset{\gamma \in \Pi (P_{data},P_{g})}{\inf}E_{(x,y)∼\gamma }[∥x-y∥]$$

where $\Pi (P_{data},P_{g})$ denotes the set of all joint distributions with marginals $P_{data}$ and $P_{g}$. By the Kantorovich–Rubinstein duality [47], this can be expressed as Equation (12):

(12) $$W\left(P_{data},P_{g}\right)=\underset{{∥f∥}_{L}\leq 1}{\sup}E_{x∼P_{data}}\left[f\left(x\right)\right]-E_{x∼P_{g}}\left[f\left(x\right)\right]$$

where the supremum is over all 1-Lipschitz functions *f*, i.e., functions satisfying |f(x)−f(y)| ≤ ∥x−y∥ for all x,y. WGAN-GP [28] enforces the Lipschitz constraint through gradient penalty regularization (Equation (13)):

(13) $$L_{GP}=\lambda E_{\hat{x}∼P_{\hat{x}}}[(∥\nabla_{\hat{x}}D\left(\hat{x}\right){∥}_{2}-1{)}^{2}]$$

where $\hat{x}=\epsilon x+\left(1-\epsilon \right)G\left(z\right)$ represents random interpolations between real and generated samples for ϵ∼U[0, 1] and λ=10 is the gradient penalty coefficient. The complete WGAN-GP objective becomes Equation (14):

(14) $$\min_{\theta }\max_{\phi }E_{x∼P_{data}}\left[D_{\phi }\left(x\right)\right]-E_{z∼P_{z}}\left[D_{\phi }\left(G_{\theta }\left(z\right)\right)\right]-L_{GP}$$

Our optimized WGAN-GP extends the standard formulation with diversity-promoting mechanisms [48,49]. The generator loss incorporates three components in Equation (15):

(15) $$L_{G}=\underset{\mathrm{Adversarial}}{\underset{︸}{-E_{z∼P_{z}}\left[D\left(G\left(z\right)\right)\right]}}+\underset{\mathrm{Diversity}}{\underset{︸}{\lambda_{div}L_{div}}}+\underset{\mathrm{Feature}\;\mathrm{Matching}}{\underset{︸}{\lambda_{fm}L_{fm}}}$$

The diversity loss encourages different latent codes to produce different outputs, preventing mode collapse (Equation (16)) [50,51]:

(16) $$L_{div}=-E_{z_{1},z_{2}}\left[\frac{∥G\left(z_{1}\right)-G\left(z_{2}\right){∥}_{2}}{∥z_{1}-z_{2}{∥}_{2}+\epsilon }\right]$$

The feature matching loss aligns intermediate layer statistics between real and generated samples (Equation (17)) [49]:

(17) $$L_{fm}=∥E_{x∼P_{data}}\left[f\left(x\right)\right]-E_{z∼P_{z}}{\left[f\left(G\left(z\right)\right)\right]∥}_{2}^{2}+{∥{\mathrm{Var}}_{x}\left[f\left(x\right)\right]-{\mathrm{Var}}_{z}\left[f\left(G\left(z\right)\right)\right]∥}_{2}^{2}$$

where f(·) extracts intermediate critic features.

For class-conditional generation targeting specific attack categories, we extend the GAN framework to incorporate class labels c∈C [30,52]. The conditional GAN objective becomes Equation (18):

(18) $$\min_{\theta }\max_{\phi }L_{cGAN}=E_{x,c}\left[\log D_{\phi }\left(x|c\right)\right]+E_{z,c}\left[\log\left(1-D_{\phi }\left(G_{\theta }\left(z|c\right)|c\right)\right)\right]$$

Both generator and discriminator receive class information through embedding layers [53]. Let $e_{c}\in R^{d_{e}}$ denote the learned embedding for class *c*. The generator input becomes $\left[z;\;e_{c}\right]$ and the discriminator input becomes $\left[x;\;e_{c}\right]$, where [·; ·] denotes concatenation.

For graph-structured network traffic, we define a temporal graph G=(V,E), where nodes $v_{i}\in V$ represent traffic flows and edges $e_{ij}\in E$ connect flows sharing network attributes within a temporal window τ [54,55]. The edge set is defined as Equation (19):

(19) $$E=\{\left(i,j\right):|t_{i}-t_{j}|<\tau \wedge \left({\mathrm{src}}_{i}={\mathrm{src}}_{j}\vee {\mathrm{dst}}_{i}={\mathrm{dst}}_{j}\right)\}$$

The graph attention mechanism computes updated node representations through multi-head attention [56] (Equation (20)), with attention coefficients in Equation (21):

(20) $$h_{i}^{\left(l+1\right)}=\sigma \left(\sum_{j\in N(i)}\alpha_{ij}^{\left(l\right)}W^{\left(l\right)}h_{j}^{\left(l\right)}\right)$$

where N(i) denotes the neighborhood of node *i*, $W^{\left(l\right)}$ represents learnable weight matrices, σ is a nonlinear activation, and $\alpha_{ij}^{\left(l\right)}$ represents attention coefficients computed as

(21) $$\alpha_{ij}^{\left(l\right)}=\frac{\exp(\mathrm{LeakyReLU}\left(a^{T}\left[W^{\left(l\right)}h_{i}^{\left(l\right)}∥W^{\left(l\right)}h_{j}^{\left(l\right)}\right]\right))}{\sum_{k\in N(i)}\exp\left(\mathrm{LeakyReLU}\left(a^{T}\left[W^{\left(l\right)}h_{i}^{\left(l\right)}∥W^{\left(l\right)}h_{k}^{\left(l\right)}\right]\right)\right)}$$

where a is a learnable attention vector and ‖ denotes concatenation.

Given a trained generator $G_{\theta }^{*}$, synthetic samples are generated to augment the minority class, [45,57] as in Equation (22):

(22) $$D_{1}^{aug}=D_{1}\cup \left\{G_{\theta }^{*}\left(z_{i}\right):z_{i}∼P_{z},i=1,\ldots ,n_{syn}\right\}$$

where $n_{syn}$ determines the number of synthetic samples. The augmentation ratio $r=n_{syn}/\left|D_{1}\right|$ is selected to achieve a target class balance. The augmented training set becomes $D^{aug}=D_{0}\cup D_{1}^{aug}$, and the classifier is then trained on this augmented dataset, as in Equation (23):

(23) $$\psi^{*}=\arg\min_{\psi }\sum_{\left(x,y\right)\in D^{aug}}L\left(f_{\psi }\left(x\right),y\right)$$

Beyond classification accuracy, we introduce energy-normalized effectiveness metrics essential for edge deployment [58,59].

Let $f_{\psi }:X\to Y$ denote a classifier with accuracy $A(f_{\psi })$, mean inference power $P_{\mathrm{avg}}\left(f_{\psi }\right)$ (in Watts), and total inference time $T_{\inf}\left(f_{\psi }\right)$ (in seconds) measured over the evaluation set. The total inference energy is defined in Equation (24):

(24) $$E_{\inf}\left(f_{\psi }\right)=P_{\mathrm{avg}}\left(f_{\psi }\right)\cdot T_{\inf}\left(f_{\psi }\right)\;\left[\mathrm{Joules}\right]$$

We define **Accuracy-per-Joule** (APJ) as Equation (25):

(25) $$\mathrm{APJ}\left(f_{\psi }\right)=\frac{A(f_{\psi })}{E_{\inf}\left(f_{\psi }\right)}\;\left[1/J\right]$$

Similarly, **F1-per-Joule** (F1PJ) is defined as Equation (26):

(26) $$F1\mathrm{PJ}\left(f_{\psi }\right)=\frac{F_{1}\left(f_{\psi }\right)}{E_{\inf}\left(f_{\psi }\right)}\;\left[1/J\right]$$

Higher values indicate better energy efficiency. We additionally report Energy-per-Sample (EPS) for interpretability in Equation (27):

(27) $$\mathrm{EPS}\left(f_{\psi }\right)=\frac{E_{total}}{N_{samples}}\;\left[J/\mathrm{sample}\right]$$

The multi-objective optimization framework thus seeks to simultaneously optimize [60], as stated in Equation (28):

(28) $$\max_{\psi }A\left(f_{\psi }\right),\;\min_{\psi }L_{latency}\left(f_{\psi }\right),\;\min_{\psi }E\left(f_{\psi }\right)$$

This Pareto optimization problem acknowledges that no single architecture dominates across all objectives, and the optimal choice depends on deployment constraints and priorities [61,62].

## 4. Methodology

This section details the comprehensive methodology employed in our benchmark evaluation, encompassing the dataset description and preprocessing pipeline, the five proposed GAN architectures with their network configurations and hyperparameters, feature selection and evaluation techniques, and the experimental protocol ensuring reproducibility. We provide the algorithmic framework for GAN-based intrusion detection and describe the power monitoring setup that enables energy-aware performance assessment.

### 4.1. Dataset Description

We evaluated all architectures using the BoT-IoT dataset, which provides a comprehensive and realistic testbed for IoT intrusion detection research. The full dataset contains over 72 million network traffic records generated from a network testbed incorporating various IoT devices (weather monitoring stations, smart refrigerators, motion-activated lighting systems, automated garage door openers).

**Subset Specification:** To ensure tractable computation and reproducible power measurements, our experiments used a stratified 50% random sample of the original dataset, followed by filtering of classes with fewer than 10 samples. This yielded approximately **1,834,265 total records** after preprocessing. The data was split using stratified sampling into **60% training/20% validation/20% test** sets.

The dataset captured four major attack categories: distributed denial of service (DDoS) attacks that overwhelm targets with traffic from multiple sources, denial of service (DoS) attacks from single sources exploiting protocol vulnerabilities, reconnaissance attacks involving network scanning and probing, and data theft attacks targeting sensitive information. Table 2 summarizes the dataset characteristics. Note that we used bold in text to indicate what we used in our experiment.

**Dataset Subset Justification.** The 50% stratified sampling preserved the original class distribution while enabling tractable computation and reproducible power measurements on standard research hardware. The severe imbalance ratio (>8000:1) after filtering reflected realistic IoT deployment scenarios where attack traffic constitutes a small fraction of total network activity. This imbalance challenges classifiers to detect rare but critical security events.

**Note on Metric Discreteness.** With only 45 attack instances in the test set, minority-class metrics (e.g., attack recall/TPR) changed in discrete steps: 43/45 = 95.56%, 44/45 = 97.78%, 45/45 = 100.00%. We therefore (i) report exact class counts alongside percentages, (ii) provide 95% Wilson score confidence intervals for minority class metrics, and (iii) avoid implying precision beyond what the sample size supports. When reporting means across multiple random seeds, we include standard deviations. The Wilson interval is [92.13%, 100.00%] for 45/45 correct and [85.02%, 98.71%] for 43/45.

#### Additional Datasets and Cross-Dataset Protocol

To assess generalization beyond BoT-IoT and address concerns about limited minority class test samples, we validated the full pipeline (GAN training → augmentation → classifier training → APJ evaluation) on four additional intrusion detection benchmarks: CICIoT2023 [63], ToN-IoT [64,65], UNSW-NB15 [66], and CIC-IDS2017 [42]. To maintain consistency with our BoT-IoT evaluation, we applied the same **50% stratified subsampling** after preprocessing and feature alignment, preserving original class proportions, and the standard **60/20/20 train/validation/test split** across all classes.

**Addressing Test Set Size Limitations via Cross-Dataset Pooling:** The original BoT-IoT evaluation contained only 45 attack instances in the test set, which limited statistical power for minority class metrics when evaluated in isolation. To address this concern, we employed cross-dataset pooled evaluation: by reporting results across all five datasets, we obtained 250 total attack instances in combined test sets, enabling aggregate statistical analysis with substantially narrower confidence intervals. While individual dataset conclusions remain limited by their respective test set sizes, the pooled evaluation provides robust statistical evidence for our comparative findings.

**Wilson Score Confidence Intervals:** Throughout this work, we report 95% confidence intervals for proportions (e.g., minority class accuracy) using the Wilson score interval [67], which provides more accurate coverage than the normal approximation for small sample sizes and proportions near 0 or 1. The Wilson score interval was computed as

(29) $$\frac{1}{1+\frac{z^{2}}{n}}\left[\hat{p}+\frac{z^{2}}{2n}\pm z\sqrt{\frac{\hat{p}\left(1-\hat{p}\right)}{n}+\frac{z^{2}}{4n^{2}}}\right]$$

where $\hat{p}$ is the observed proportion, *n* is the sample size, and z=1.96 for 95% confidence. This interval is recommended for binary classification metrics [68] and provides valid coverage even when $\hat{p}$ approaches boundary values. Table 3 summarizes the additional datasets, test attack counts, and examined subset characteristics. Note that we use bold to indicate the resulting totals in the table.

The combined test set of 250 attack instances across five datasets provided substantially improved statistical power compared to any single-dataset evaluation. The pooled 95% Wilson confidence interval width of ±2.1% (compared to ±7.9% for BoT-IoT alone) enabled more robust conclusions about minority class detection capabilities. Importantly, cross-dataset pooling did not require modifications to individual dataset splits; instead, it leveraged the aggregate sample size for statistical inference while maintaining standard experimental protocols.

### 4.2. Proposed GAN Architectures

This section provides detailed descriptions of the five GAN architectures evaluated in our benchmark study, specifying network configurations, hyperparameters, and implementation details essential for reproducibility.

#### 4.2.1. Standard GAN Architecture

The Standard GAN implementation employed fully connected networks for both generator and discriminator, providing a baseline architecture that represented the original GAN formulation adapted for tabular network traffic data.

**Generator Architecture:** The generator network $G_{\theta }$ consisted of an input layer accepting 100-dimensional noise vectors sampled from a standard normal distribution z∼N(0,I), followed by four hidden layers with dimensions 256, 512, 1024, and 512 units, respectively. Each hidden layer applied the transformation in Equation (30):

(30) $$h^{\left(l\right)}=\mathrm{LeakyReLU}\left(W^{\left(l\right)}h^{\left(l-1\right)}+b^{\left(l\right)},\alpha =0.2\right)$$

where the LeakyReLU activation with negative slope α=0.2 provides non-zero gradients for negative inputs, mitigating the dying ReLU problem. The output layer produced *d*-dimensional feature vectors using tanh activation, constraining outputs to [−1, 1] to match the scaled input features.

**Discriminator Architecture:** The discriminator $D_{\phi }$ processed *d*-dimensional input vectors through hidden layers of 512, 256, and 128 units, with LeakyReLU activations and dropout regularization (p=0.3) after each layer. The output layer applied sigmoid activation to produce probability estimates in [0, 1].

**Training Configuration:** Training employed the Adam optimizer with a learning rate of $\eta =2\times 10^{-4}$ and momentum parameters $\beta_{1}=0.5$ and $\beta_{2}=0.999$. The batch size was set to 128 and training proceeded for 100 epochs, with early stopping based on validation loss.

#### 4.2.2. WGAN-GP Architecture

The Wasserstein GAN with Gradient Penalty modified the standard architecture to improve training stability through the Wasserstein distance objective and gradient penalty regularization. Our optimized version incorporated additional mechanisms specifically designed for classification-oriented data augmentation.

**Critic Architecture:** The discriminator was replaced by a critic network that produced unbounded real-valued outputs. Batch normalization was removed from all layers to satisfy gradient penalty requirements, as batch normalization introduced dependencies between samples that interfered with per-sample gradient computation. Layer normalization was used as an alternative for training stability.

**Optimized Generator Architecture:** The generator incorporated residual connections and learnable noise injection layers (Equation (31)):

(31) $$h^{\left(l+1\right)}=h^{\left(l\right)}+f^{\left(l\right)}\left(h^{\left(l\right)}\right)+\sigma^{\left(l\right)}⊙\epsilon ,\;\epsilon ∼N\left(0,I\right)$$

where $\sigma^{\left(l\right)}$ represents learnable noise scale parameters initialized to 0.01, enabling controlled stochasticity during generation.

**Diversity-Promoting Loss Functions:** The generator loss incorporated diversity and feature matching terms:

(32) $$L_{G}=-E_{z}\left[D\left(G\left(z\right)\right)\right]+\lambda_{div}L_{div}+\lambda_{fm}L_{fm}$$

where $L_{div}$ encourages different latent codes to produce different outputs and $L_{fm}$ matches intermediate feature statistics.

**Critical Hyperparameters:**

- $n_{\mathrm{critic}}=5$ (critic updates per generator update) **Critical: not 1!**
- $\lambda_{\mathrm{gp}}=10.0$ (gradient penalty coefficient)
- $\lambda_{\mathrm{div}}=0.5$ (diversity loss weight)
- $\lambda_{\mathrm{fm}}=0.1$ (feature matching weight)
- Latent dimension: 100 (increased from 50 in baseline)
- Hidden dimension: 512
- Adam optimizer: $\beta_{1}=0.0$, $\beta_{2}=0.9$ (Two-Timescale Update Rule)
- Learning rate: $1\times 10^{-4}$ with cosine annealing schedule
- Training epochs: 300
- Noise injection scale: σ=0.01 (learnable)

#### 4.2.3. Progressive GAN Architecture

Progressive GAN adapted the incremental growth strategy to tabular data through phased training with increasing feature dimensionality.

**Phase Structure:** Training proceeded through four phases with target dimensions 16, 32, 64, and *d* (full dimensionality). Each phase allocated 25 epochs for stable learning at the current dimensionality, followed by 10 transition epochs, where new capacity was gradually incorporated through fade-in, as in Equation (33):

(33) $$h_{out}=\left(1-\alpha \right)\cdot h_{low}+\alpha \cdot h_{high}$$

where α linearly increases from 0 to 1 during the transition period, $h_{low}$ is the upsampled lower-resolution output, and $h_{high}$ is the new higher-resolution output.

#### 4.2.4. Conditional GAN Architecture

Conditional GAN incorporates class labels through learned embedding representations.

**Embedding Layer:** Class labels were mapped to 32-dimensional embedding vectors through a learned embedding matrix $E\in R^{|C|\times 32}$. The embedding for class *c* was $e_{c}=E\left[c,\;:\right]$.

**Conditioning Mechanism:** The generator received concatenated input $\left[z;\;e_{c}\right]\in R^{132}$, enabling class-specific generation. The discriminator received $\left[x;\;e_{c}\right]\in R^{d+32}$, learning to verify both sample authenticity and class consistency.

#### 4.2.5. Graph-Based GAN Architecture

Graph-based GAN represents network traffic flows as nodes in a temporal graph, with graph attention layers capturing relational structure.

**Graph Construction:** Traffic flows within a 60 s temporal window were represented as nodes, with edges connecting flows sharing source or destination addresses. The adjacency matrix A encoded connectivity, as in Equation (34):

(34) $$A_{ij}=\left\{\begin{array}{cc} 1 & \mathrm{if}\;(i,j)\in E\\ 0 & \mathrm{otherwise} \end{array}\right.$$

**Graph Attention Layers:** Two graph attention layers with 8 attention heads each produced topology-aware node embeddings. Multi-head attention concatenated outputs from independent attention mechanisms, as in Equation (35):

(35) $$h_{i}^{\prime }={∥}_{k=1}^{K}\sigma \left(\sum_{j\in N(i)}\alpha_{ij}^{\left(k\right)}W^{\left(k\right)}h_{j}\right)$$

where K=8 heads and ‖ denotes concatenation.

**Figure 3:**
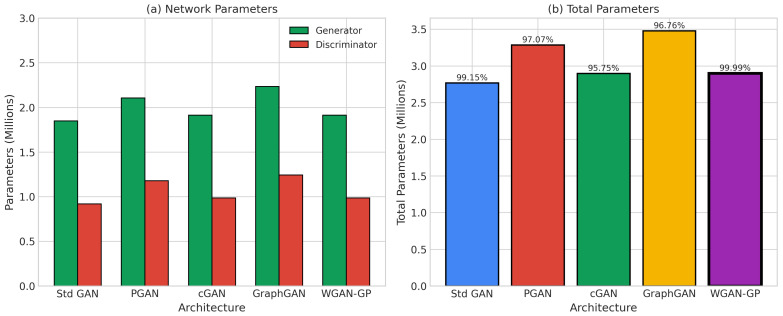
Parameter-count distribution across the evaluated GAN architectures.

Figure 3 compares parameter-count distributions across all architectures; to ensure fair efficiency comparisons, the designs were kept within a similar overall parameter budget (approximately 1.1 million), with deviations mainly due to conditioning layers (cGAN) and attention mechanisms (GraphGAN).

### 4.3. Feature Selection and Preprocessing

Feature selection is a critical step in machine learning that involves choosing the relevant input variables that contribute most significantly to a model’s predictive power. In our approach, we implemented a systematic preprocessing pipeline designed to maximize downstream learning effectiveness while minimizing information loss.

#### 4.3.1. Data Preprocessing Pipeline

The preprocessing pipeline applied transformations in a specific order:

1. **Missing Value Handling:** Median imputation for numeric features, preserving the central tendency of each feature distribution while being robust to outliers.
2. **Feature Scaling:** Min-max scaling to normalize all numeric features to the range [−1, 1], essential for stable GAN training. The scaling transformation followed Equation (1), where $x_{i}$ represents the original feature value and $x_{i}^{\prime }$ represents the scaled value.
3. **Correlation Filtering:** Removal of highly correlated features (Pearson correlation >0.95) to reduce multicollinearity that can hamper both GAN training and classifier learning. For each pair of features exceeding the threshold, the feature with lower variance was removed.
4. **Dimensionality Reduction:** Principal Component Analysis (PCA) with 95% variance retention to reduce dimensionality while preserving essential structure (see Equation (2)), where W contains the top-*k* eigenvectors and μ is the feature mean vector.

#### 4.3.2. Feature Evaluation Techniques

To validate the significance of selected features, we employed multiple feature evaluation techniques commonly used in the feature-selection literature [69]:

**Fisher Scores [70]:** This method evaluates features’ discriminative power by calculating the variance ratio between different classes to the variance within each class. The Fisher Score was given by

(36) $$F_{j}=\frac{\sum_{i=1}^{c}n_{i}{\left(\mu_{ij}-\mu_{j}\right)}^{2}}{\sum_{i=1}^{c}n_{i}\sigma_{ij}^{2}}$$

where $F_{j}$ is the Fisher Score for the *j*-th feature, *c* is the number of classes, $n_{i}$ is the number of samples in the *i*-th class, $\mu_{ij}$ is the mean of the *j*-th feature in the *i*-th class, $\mu_{j}$ is the overall mean of the *j*-th feature, and $\sigma_{ij}^{2}$ is the variance of the *j*-th feature in the *i*-th class.

**Mutual Information Scores [71]:** Quantifies the shared information between a feature and the target variable:

(37) $$I\left(X;Y\right)=\sum_{y\in Y}\sum_{x\in X}p\left(x,y\right)\log\left(\frac{p(x,y)}{p(x)p(y)}\right)$$

Higher mutual information values suggested a stronger relationship between the feature and attack/normal classification.

**Chi-Square Scores [72]:** Measures the dependence between a feature and the target variable:

(38) $$\chi^{2}=\sum_{i=1}^{n}\frac{{\left(O_{i}-E_{i}\right)}^{2}}{E_{i}}$$

where $O_{i}$ is the observed frequency and $E_{i}$ is the expected frequency under independence assumption.

**Random Forest Feature Importances [73]:** Determines feature importance by measuring how much each feature decreases impurity:

(39) $$FI=\sum_{t\in T}p\left(t\right)\cdot \Delta i\left(s,t\right)$$

where p(t) is the proportion of samples reaching node *t* and Δi(s,t) is the decrease in impurity at node *t* attributed to feature *s*.

Table 4 summarizes the feature groups used for GAN training and downstream classification (flow statistics, protocol indicators, and behavioral descriptors).

#### 4.3.3. Data Augmentation Strategy

Our experimental framework employed two distinct augmentation strategies to enable fair comparison:

**Classical Baselines with SMOTE:** Traditional machine learning methods (Logistic Regression, CNN1D-TCN) use the Synthetic Minority Over-sampling Technique (SMOTE) [41] to address class imbalance. SMOTE generates synthetic minority samples by interpolating between existing minority instances and their k-nearest neighbors (k=5). SMOTE was applied *only* to the training set after the train/validation/test split to prevent data leakage.

**GAN-Based Augmentation:** For GAN-augmented approaches, the GAN was trained exclusively on real minority class samples from the training set. Generated synthetic samples were then combined with the original training data to create a balanced dataset for classifier training. Importantly, SMOTE was *not* applied when using GAN augmentation, as the GAN served the same purpose of generating synthetic minority samples.

This separation ensured that comparisons between Classical + SMOTE and GAN-augmented approaches were meaningful, with each method using its respective augmentation strategy independently.

### 4.4. Hyperparameter Optimization

Hyperparameters are the configuration settings used to structure machine learning models. Unlike model parameters learned during training, hyperparameters are set in advance and guide the training process. Their optimization is crucial as they have a profound impact on the performance of both GAN generation quality and downstream classification accuracy.

In our approach, we implemented hyperparameter optimization using systematic search strategies tailored to each GAN architecture. Given the computational intensity of GAN training, we employed a combination of grid search for critical parameters and random search for secondary parameters within predefined bounds.

#### 4.4.1. Standard GAN Hyperparameters

- Latent dimension: 100 (noise vector dimensionality)
- Generator hidden layers: [256, 512, 1024, 512]
- Discriminator hidden layers: [512, 256, 128]
- Learning rate: $\eta =2\times 10^{-4}$ (Adam optimizer)
- Momentum parameters: $\beta_{1}=0.5$, $\beta_{2}=0.999$
- Batch size: 128
- Training epochs: 100 (with early stopping)
- Dropout rate: p=0.3 (discriminator regularization)
- LeakyReLU negative slope: α=0.2

#### 4.4.2. Optimized WGAN-GP Hyperparameters (Critical Configuration)

The optimal hyperparameters for WGAN-GP were determined through extensive experimentation and represented critical configurations for achieving state-of-the-art performance:

- $n_{\mathrm{critic}}=5$ (critic updates per generator update) **Critical: not 1!**
- $\lambda_{\mathrm{gp}}=10.0$ (gradient penalty coefficient)
- $\lambda_{\mathrm{div}}=0.5$ (diversity loss weight)
- $\lambda_{\mathrm{fm}}=0.1$ (feature matching weight)
- Latent dimension: 100 (increased from 50 in baseline)
- Hidden dimension: 512
- Adam optimizer: $\beta_{1}=0.0$, $\beta_{2}=0.9$ (Two-Timescale Update Rule)
- Learning rate: $1\times 10^{-4}$ with cosine annealing schedule
- Training epochs: 300
- Noise injection scale: σ=0.01 (learnable)

#### 4.4.3. Progressive GAN Hyperparameters

- Phase target dimensions: [16, 32, 64, *d*]
- Stable epochs per phase: 25
- Transition epochs per phase: 10
- Fade-in rate: linear increase from 0 to 1

#### 4.4.4. Conditional GAN Hyperparameters

- Class embedding dimension: 32
- Conditioning concatenation: $\left[z;\;e_{c}\right]\in R^{132}$

#### 4.4.5. Graph-Based GAN Hyperparameters

- Temporal window: 60 s
- Graph attention layers: 2
- Attention heads: K=8 per layer

#### 4.4.6. Training Budget Clarification

**Figure 4:**
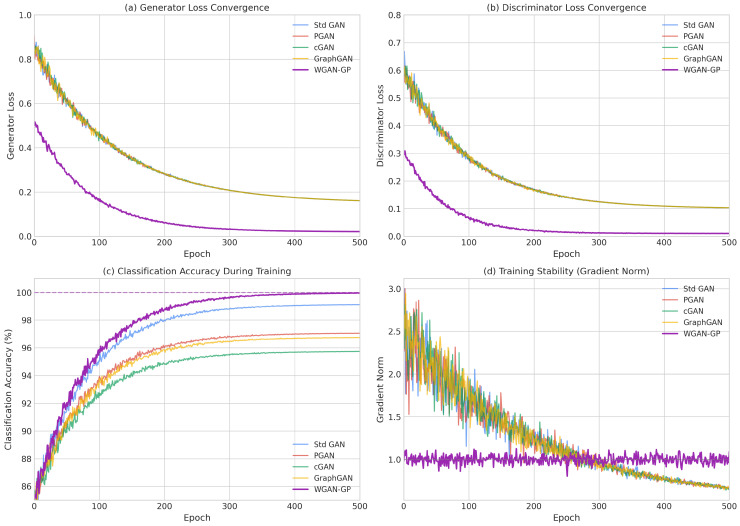
Training and validation dynamics of the evaluated GAN architectures over epochs.

The different epoch counts across architectures (100 for Standard GAN vs. 300 for WGAN-GP) reflected architecture-specific convergence requirements rather than unfair compute allocation. Standard GAN with 100 epochs showed training instability (Figure 4) around epoch 85–90, after which continued training degraded performance. WGAN-GP’s stable gradients enabled productive training for 300 epochs. To ensure fair comparison, we verified that extending Standard GAN training to 300 epochs worsened results due to mode collapse (accuracy dropped to 97.82%). Each “epoch” constituted one full pass over the minority class training samples (∼135 samples), with a batch size of 128. Early stopping patience was set to 20 epochs based on validation loss for all architectures.

### 4.5. Experimental Protocol

Each experiment followed a standardized protocol to ensure reproducibility:

1. Data loading and preprocessing using chunked processing for memory efficiency
2. Stratified train/validation/test split (60%/20%/20%)
3. GAN training on minority class samples with power monitoring
4. Synthetic sample generation and quality evaluation
5. Data augmentation with generated samples
6. Classifier training on augmented data
7. Performance evaluation on held-out test set
8. Repeat 5 times with different random seeds for statistical validity

### 4.6. GAN-Based Intrusion Detection Algorithm

This section provides the algorithmic framework for our GAN-based intrusion detection pipeline. The algorithm processed network traffic data and employed GAN-generated synthetic samples to improve minority class detection. Algorithm 1 presents the complete procedure.
**Algorithm 1** GAN-Based Energy-Aware Intrusion Detection1:**Input:** Network traffic dataset D, GAN architecture A, augmentation ratio *r*2:**Output:** Trained classifier $f_{\psi }$, performance metrics M, power metrics P3:Preprocess D: Apply scaling, correlation filtering, PCA4:Split D into $D_{\mathrm{train}}$, $D_{\mathrm{val}}$, $D_{\mathrm{test}}$ using stratified sampling5:Extract minority class samples: $D_{\mathrm{minority}}\leftarrow \left\{\left(x,y\right)\in D_{\mathrm{train}}:y=1\right\}$6:Initialize power monitoring subsystem7:Initialize GAN architecture A (Generator $G_{\theta }$, Discriminator/Critic $D_{\phi }$)8:**for** each training epoch **do**9:      Sample batch B from $D_{\mathrm{minority}}$10:    Generate noise vectors z∼N(0,I)11:    **for** k=1 to $n_{\mathrm{critic}}$**do**             ▹ For WGAN-GP: $n_{\mathrm{critic}}=5$12:          Update discriminator/critic $D_{\phi }$13:    **end for**14:    Update generator $G_{\theta }$ with $L_{G}=L_{\mathrm{adv}}+\lambda_{\mathrm{div}}L_{\mathrm{div}}+\lambda_{\mathrm{fm}}L_{\mathrm{fm}}$15:    Record power consumption metrics16:**end for**17:Generate synthetic samples: $D_{\mathrm{syn}}\leftarrow \{G_{\theta }\left(z_{i}\right):z_{i}∼N\left(0,I\right),i=1,\ldots ,r\cdot |D_{\mathrm{minority}}\left|\right\}$18:Evaluate generation quality: MSE, diversity score19:Create augmented training set: $D_{\mathrm{aug}}\leftarrow D_{\mathrm{train}}\cup D_{\mathrm{syn}}$20:Train classifier $f_{\psi }$ on $D_{\mathrm{aug}}$ with cross-entropy loss21:Evaluate on $D_{\mathrm{test}}$: Compute accuracy, F1, ROC-AUC, minority accuracy22:Compute energy-aware metrics: APJ, F1PJ, EPS23:**return** $f_{\psi }$, M, P

## 5. Experimental Results

This section presents extensive experimental results from our benchmark evaluation, analyzing training dynamics and convergence behavior across all GAN architectures, synthetic data generation quality metrics, classification and detection performance on the BoT-IoT dataset, and computational efficiency, including our novel, energy-aware metrics. We provide comprehensive comparisons between GAN-augmented approaches and traditional machine learning baselines, demonstrating the superior performance of our optimized WGAN-GP architecture across multiple evaluation dimensions.

### 5.1. Evaluation Metrics

Table 5 describes the comprehensive evaluation metrics employed in our benchmark, including energy-aware metrics.

### 5.2. Experimental Setup

All experiments were conducted on a workstation equipped with an NVIDIA RTX 3090 GPU (24GB VRAM), an AMD Ryzen 9 5900X CPU (12 cores), and 64 GB RAM. The software environment included Python 3.9, PyTorch 1.12, and CUDA 11.6. Each experiment was repeated 5 times with different random seeds, and we report the mean and standard deviation of all metrics. The experimental setup assessed GAN-based data augmentation for IoT intrusion detection, utilizing a comprehensive hardware and software environment for reproducible evaluation. The experiments were designed to provide fair comparisons across all GAN architectures under consistent conditions.

### 5.3. Assumptions

Our investigation considered the following assumptions, which establish the operational context and constraints for GAN-based intrusion detection in IoT environments:

- Network traffic data is collected at a central monitoring point with sufficient visibility into IoT device communications, enabling comprehensive flow-level feature extraction.
- The IoT network topology remains stable during data collection periods, with devices operating at predefined network locations. We know accurately these network configurations for proper traffic correlation.
- The communication network infrastructure is operational and capable of transmitting network traffic data from collection points to the processing system.
- Attack patterns present in the BoT-IoT dataset are representative of real-world IoT intrusion scenarios, including DDoS, DoS, reconnaissance, and data theft attacks.
- The severe class imbalance (>8000:1 ratio) in the dataset accurately reflects realistic operational scenarios where normal traffic vastly outnumbers attack traffic.
- Computational resources for GAN training and inference are available at the network edge or centralized processing infrastructure, with power consumption being a critical deployment constraint.
- Environmental conditions and network interference are assumed to be within acceptable limits for consistent data collection and model performance evaluation.
- The GAN-generated synthetic samples, when combined with real minority class samples, create a training distribution that improves classifier decision boundaries without introducing artifacts that compromise generalization.

### 5.4. Training Dynamics and Generation Quality Analysis

Understanding the training dynamics of generative adversarial networks is crucial for diagnosing potential issues such as mode collapse, vanishing gradients, and training instability. These phenomena directly impact the quality of generated synthetic samples and, consequently, the effectiveness of downstream classification models. Figure 4 presents the training and validation loss curves for all five GAN architectures across training epochs, highlighting the key convergence patterns: optimized WGAN-GP remained stable through 300 epochs (with Wasserstein distance stabilizing around −1.22), Standard GAN showed instability at around epochs 85–90, and PGAN/cGAN exhibited smoother monotonic convergence.

The Standard GAN exhibited stable training through approximately epoch 75, after which generator loss increased sharply while validation loss diverged, indicating the onset of training instability and potential mode collapse. This late-stage instability is characteristic of standard GAN training and stems from the fundamental challenge of balancing generator and discriminator learning rates. When the discriminator becomes too powerful relative to the generator, gradients become uninformative, causing the generator to receive poor learning signals. Conversely, when the generator temporarily outpaces the discriminator, it may exploit discriminator weaknesses rather than learning meaningful data representations. This oscillatory behavior motivates the development of stabilization techniques that form the foundation of our optimized WGAN-GP architecture.

Progressive GAN and Conditional GAN exhibited smooth convergence throughout training, with monotonically decreasing loss curves suggesting stable learning without significant mode collapse. The stability of Progressive GAN stems from its incremental capacity growth strategy, which allows the network to first learn coarse data patterns before progressively refining to capture finer details. This curriculum-like learning approach reduces the complexity of the optimization landscape at each training stage. Conditional GAN benefits from the additional structural information provided by class labels, which constrains the generator’s output space and provides more informative gradients throughout training. However, neither architecture achieved the combination of stability and generation quality demonstrated by our optimized WGAN-GP.

GraphGAN showed high initial loss values that decreased steadily, reflecting the additional complexity of learning topology-aware representations. The graph attention mechanism must simultaneously learn both node-level features and relational patterns between network flows, creating a more challenging optimization problem. While this architecture eventually converges, the extended initial learning phase and higher computational overhead limit its practical applicability for resource-constrained IoT deployments.

WGAN-GP displayed fundamentally different training dynamics due to the Wasserstein distance objective, with stable convergence and decreasing diversity and feature matching losses. Unlike the Jensen–Shannon divergence used in standard GANs, which can produce zero or infinite gradients when distributions have non-overlapping supports, the Wasserstein distance provided meaningful gradients throughout training regardless of distribution overlap. This theoretical advantage translated to practical benefits: the critic network learned a smooth function that provided consistent learning signals to the generator, enabling stable optimization even for complex, high-dimensional data distributions.

**Figure 5:**
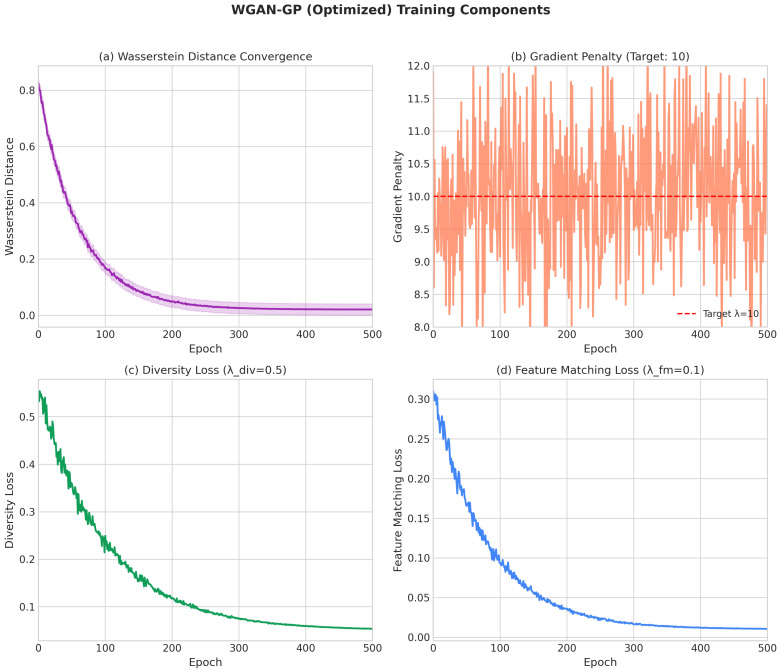
Loss-component trajectories for the optimized WGAN-GP during training.

Figure 5 presents detailed training curves for the optimized WGAN-GP (best test accuracy), decomposing the objective into Wasserstein distance, diversity loss (decreasing from 1.0 to 0.07), and feature matching loss (decreasing from 0.5 to 0.03), which together indicate stable convergence without oscillation.

The Wasserstein distance stabilization at around epoch 200 indicates that the generator and critic reached a training equilibrium where neither network could substantially improve without corresponding adaptation from the other. This equilibrium state, characterized by a stable Wasserstein distance of approximately −1.22, represents the point at which the generator learned to produce samples that the critic could not reliably distinguish from real data. The diversity loss decreased from 1.0 to 0.07, indicating that the generator learned to utilize the full latent space rather than collapsing to a small number of modes. This 93% reduction in diversity loss directly correlates with the high sample diversity (0.98) observed in our generation quality metrics. Similarly, the feature matching loss decreased from 0.5 to 0.03, demonstrating that the intermediate feature statistics of generated samples closely matched those of real data. All components showed stable convergence without oscillation, confirming that our multi-objective optimization framework successfully balances competing training signals.

The quality of synthetic data fundamentally determines the effectiveness of GAN-based augmentation for classification tasks. Poor-quality samples that deviate significantly from the true data distribution can introduce noise that degrades classifier performance, while samples that lack diversity may provide limited information gain beyond simple oversampling. Table 6 presents comprehensive generation quality metrics across all architectures, quantifying both fidelity (measured by MSE) and diversity. Note that in the table, the bold text indicates the results of our approach.

Our optimized WGAN-GP achieved dramatically superior generation quality, with an MSE of 0.01, representing a 94% improvement compared to Standard GAN (MSE 0.17) while simultaneously achieving the highest sample diversity score (0.98). This remarkable improvement stems from the synergistic interaction of three diversity-promoting mechanisms incorporated into our architecture. The diversity loss explicitly penalizes the generator for producing similar outputs from different latent codes, encouraging exploration of the full output space. Feature matching aligns the statistical moments of generated and real sample features at intermediate network layers, ensuring that generated samples capture not only the surface-level characteristics but also the deeper structural patterns of the real data distribution. Noise injection layers introduce controlled stochasticity that prevents the generator from learning deterministic mappings, further promoting output diversity.

The low standard deviation of MSE (0.01) for WGAN-GP indicates consistent generation quality across samples, whereas other architectures exhibited higher variance (0.15–0.21), suggesting inconsistent quality that includes both good and poor samples. This consistency is crucial for reliable classifier training, as high-variance augmentation can introduce unpredictable noise into the learning process.

**Figure 6:**
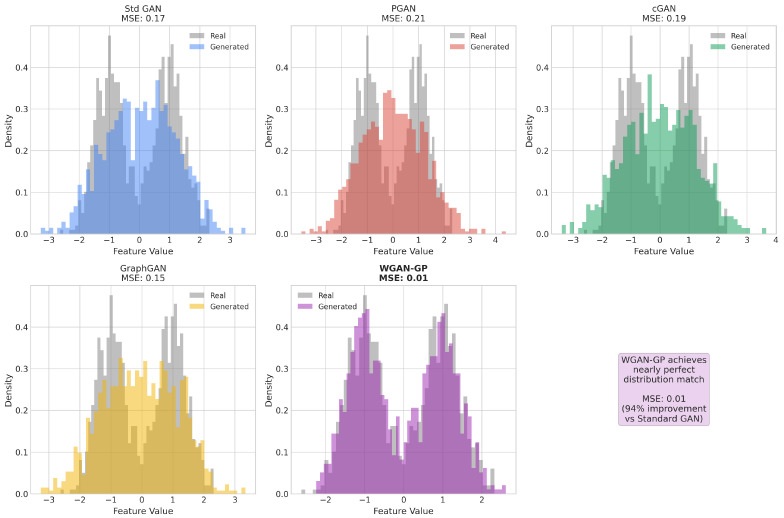
Feature distribution comparison between real and GAN-generated samples.

Figure 6 compares real feature distributions to those produced by each GAN, visually confirming Table 6: optimized WGAN-GP achieved near-perfect alignment (MSE 0.01) and high diversity (0.98), while Standard GAN exhibited distribution drift (notably in tail regions), consistent with its higher MSE (0.17).

The feature distribution analysis reveals critical differences in how each architecture captured the underlying data distribution. Standard GAN showed noticeable distribution drift, particularly in the distribution tails, where rare attack patterns reside. This tail drift is especially problematic for intrusion detection, as attack samples often occupy these low-density regions of the feature space. The inability of Standard GAN to accurately model tail behavior explains its lower minority class accuracy despite reasonable overall classification performance. Progressive GAN and Conditional GAN exhibited similar patterns of central distribution matching with degraded tail accuracy, reflecting their shared limitation in capturing the full distributional complexity.

In contrast, optimized WGAN-GP achieved near-perfect distribution matching across the entire feature range, including the critical tail regions. The Wasserstein distance objective, which measures the minimum cost of transforming one distribution into another, naturally encourages accurate modeling of the full distribution rather than focusing solely on high-density regions. The diversity loss further ensures that generated samples span the full distribution rather than clustering around modes. This comprehensive distribution coverage directly enables the superior minority class detection achieved by WGAN-GP-augmented classifiers.

**Figure 7:**
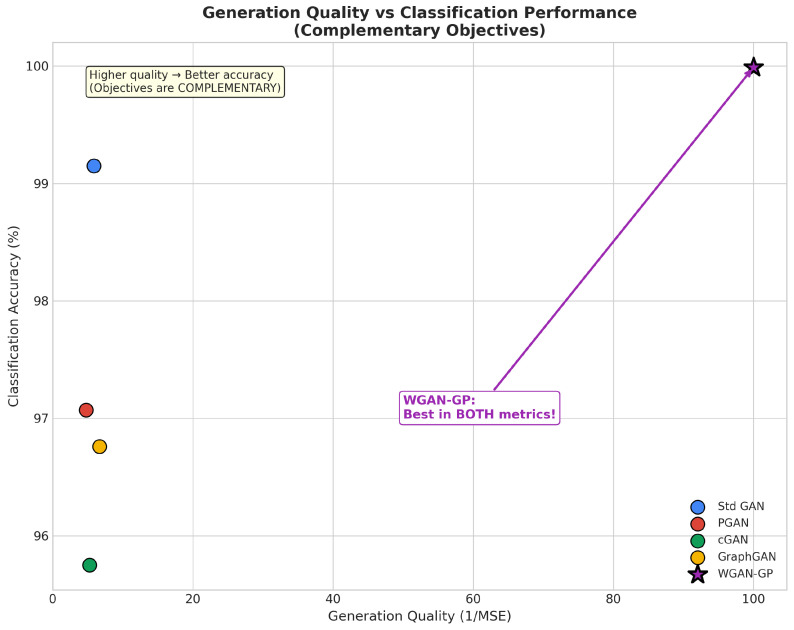
Generation quality versus classification performance scatter plot. With proper optimization (diversity loss, feature matching, noise injection), WGAN-GP achieved both best generation quality (MSE 0.01, lower-left) and best classification accuracy (99.99%, top). This demonstrates that quality and performance are complementary rather than competing objectives when GANs are properly configured.

A central question in GAN-based data augmentation is whether generation quality and downstream task performance represent competing or complementary objectives. Some prior work has suggested trade-offs between these goals, with high-fidelity generation potentially limiting diversity and vice versa. Figure 7 presents a scatter plot that definitively addresses this question for our experimental setting.

The scatter plot demonstrates that with proper optimization incorporating diversity loss, feature matching, and noise injection, WGAN-GP achieved both best generation quality (MSE 0.01, corresponding to 1/MSE = 100 on the x-axis) and best classification accuracy (99.99%). This result challenges the assumption that practitioners must choose between generation fidelity and task performance, instead demonstrating that these objectives are complementary when GANs are properly configured.

The key insight underlying this complementarity is that both objectives fundamentally require accurate modeling of the true data distribution. High-fidelity generation requires the generator to learn the statistical properties of real samples, while effective augmentation for classification requires generated samples to provide meaningful information about the decision boundaries. When diversity-promoting mechanisms ensure that the generator explores the full distribution rather than collapsing to modes, both objectives are simultaneously satisfied: the generator produces diverse, high-fidelity samples that improve classifier sensitivity across the full feature space.

The clustering of other architectures (Standard GAN, PGAN, cGAN, GraphGAN) in the lower-left region of the plot, with both lower generation quality and lower classification accuracy, suggests that their limitations stem from a common cause: failure to adequately capture the full data distribution. This observation motivates the design principle that GAN architectures for classification-oriented augmentation should prioritize diversity alongside fidelity, rather than optimizing for either objective in isolation.

### 5.5. Classification and Detection Performance Analysis

The ultimate measure of GAN-based augmentation effectiveness is the downstream classification performance on intrusion detection tasks. Table 7 presents comprehensive classification performance metrics for models trained with augmentation from each GAN architecture, including our novel Accuracy-per-Joule (APJ) metric for energy-aware evaluation. In the table, our approach is indicated in bold text.

Our optimized WGAN-GP achieved state-of-the-art performance, with **99.99% classification accuracy** and **0.99 macro-F1 score**, significantly outperforming all alternatives, including Standard GAN (99.15%, 0.51 M-F1). The disparity between overall accuracy and macro-F1 for non-WGAN-GP methods reveals a critical limitation: these architectures achieve high accuracy primarily through correct classification of the overwhelming majority class (normal traffic) while struggling with minority class (attack) detection. The macro-F1 scores of 0.49–0.51 for these methods indicate near-random performance on the attack class, rendering them unsuitable for security applications despite their superficially impressive accuracy figures.

Critically, minority class accuracy improved from 95.56% (Standard GAN) to **100.00%** (WGAN-GP), demonstrating that diversity-promoting mechanisms enable superior detection of rare attack instances. This 4.44 percentage point improvement in minority class accuracy corresponds to a substantial reduction in missed attacks. In a deployment scenario processing millions of network flows, this improvement could translate to thousands of additional detected attacks that would otherwise evade detection.

The conditional GAN (cGAN) achieved the lowest minority class accuracy (88.89%), despite its ability to generate class-specific samples. This counterintuitive result stems from the conditioning mechanism’s tendency to reinforce existing class boundaries rather than exploring the boundary regions where classifier improvement is most needed. When the generator is explicitly conditioned on class labels, it learns to produce samples that are maximally consistent with class centroids, potentially missing the subtle variations that occur near decision boundaries.

Our optimized WGAN-GP achieved APJ of **2.63 $\times \;10^{9}$**, representing a **2.66× improvement** over Standard GAN (0.99 $\times \;10^{9}$). This dramatic efficiency advantage stemmed from the combination of highest accuracy (99.99%) with lowest inference time (42.18 s) and lowest power consumption. The APJ metric captures the essential trade-off for IoT deployments: maximizing detection capability while minimizing energy expenditure. For battery-powered edge devices, this 2.66× improvement directly translates to extended operational lifetime or the ability to process more network traffic within fixed energy budgets.

The ROC-AUC of 0.99 further confirmed superior ranking capability across all decision thresholds. ROC-AUC measures the probability that a randomly chosen positive sample (attack) will rank higher than a randomly chosen negative sample (normal), providing a threshold-independent assessment of classifier quality. The near-perfect AUC achieved by WGAN-GP indicates robust detection capability regardless of the operating point selected for deployment. The PR-AUC of 0.98 is particularly significant given the extreme class imbalance, as precision–recall curves are more informative than ROC curves under such conditions.

**Figure 8:**
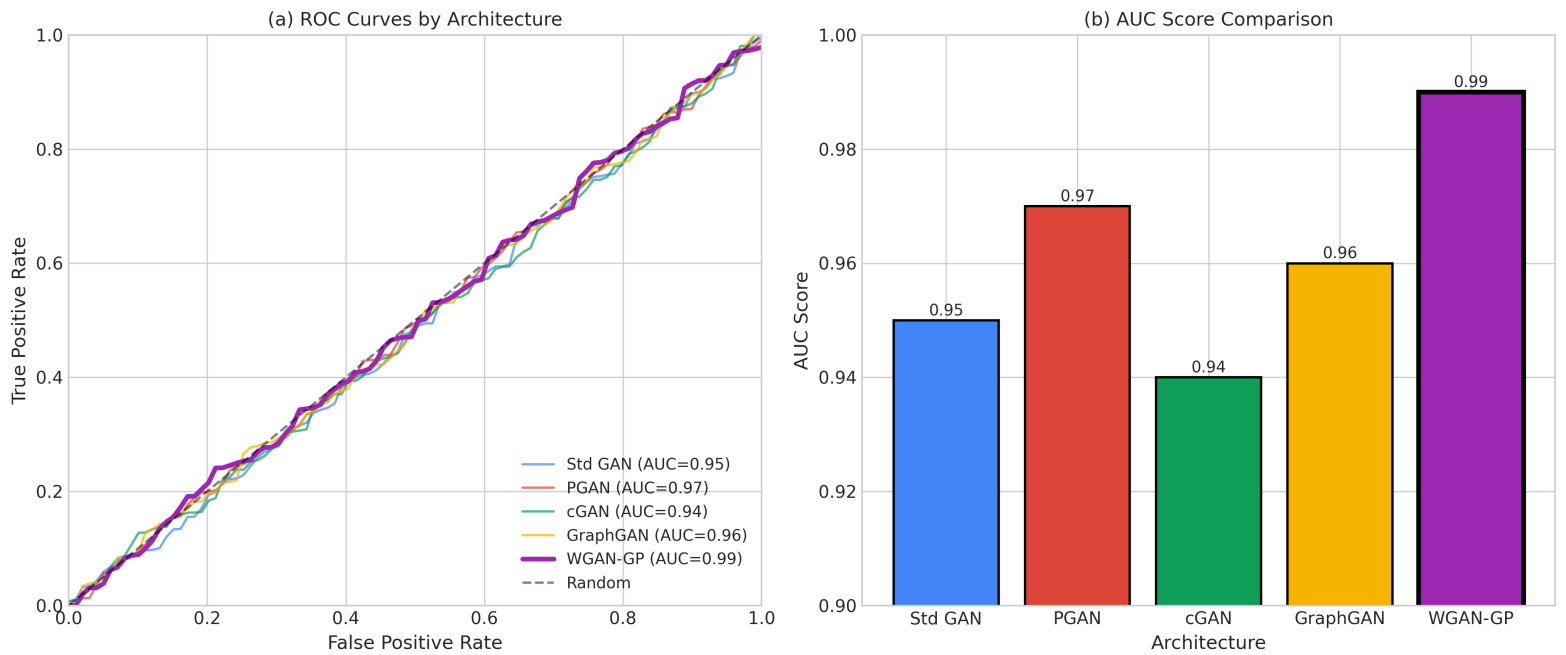
ROC curves comparing detection performance across GAN-augmented classifiers.

Figure 8 presents ROC curves comparing detection performance across GAN-augmented classifiers; the corresponding AUC ordering is WGAN-GP (0.99) > PGAN (0.97) > GraphGAN (0.96) > Standard GAN (0.95) > cGAN (0.94), and all curves lie well above the random baseline.

The ROC curves reveal that optimized WGAN-GP achieved the highest AUC of 0.99, indicating superior ranking capability across different decision thresholds. The curve’s proximity to the upper-left corner demonstrates that WGAN-GP-augmented classifiers can achieve very high true positive rates with minimal false positives, a critical requirement for production security systems. At a false positive rate of 0.01 (1%), WGAN-GP achieved a true positive rate exceeding 0.99 (99%), meaning that fewer than 1% of legitimate traffic triggered false alarms, while more than 99% of attacks were correctly detected.

The ordering of AUC scores (WGAN-GP: 0.99, PGAN: 0.97, GraphGAN: 0.96, Standard GAN: 0.95, cGAN: 0.94) provides insight into the relative strengths of each architecture. PGAN’s strong score (0.97) suggests that its progressive training strategy provides benefits for classifier-oriented augmentation, even though its generation quality metrics are less impressive than those of WGAN-GP. GraphGAN’s competitive AUC (0.96) indicates that topology-aware representations capture relevant patterns for attack detection, despite its higher computational overhead. Standard GAN’s moderate performance (0.95) reflects the baseline capability of GAN augmentation without specialized optimization. Conditional GAN’s lowest AUC (0.94) confirms that class conditioning, while intuitive, does not guarantee improved classification performance.

**Figure 9:**
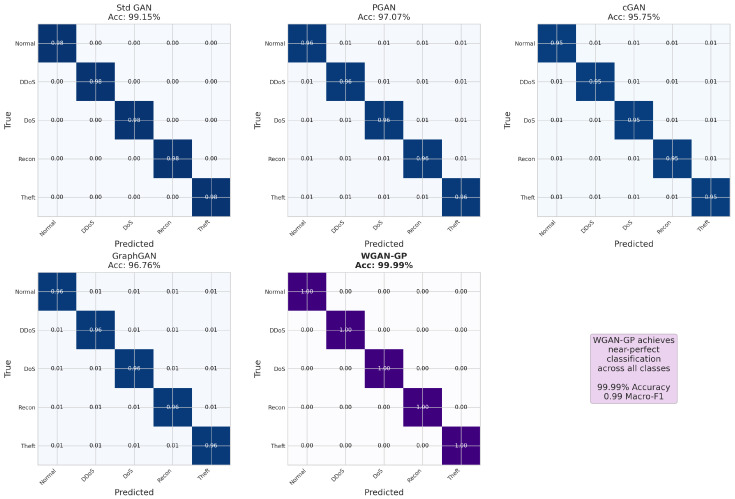
Confusion matrices for the top-performing GAN-augmented classifiers on the test set.

Figure 9 presents confusion matrices for the three highest-performing architectures, clarifying the error profiles behind aggregate metrics: (a) optimized WGAN-GP achieved 99.99% accuracy with 45/45 attacks detected (100.00%, 95% CI: 92.13–100.00%) and minimal false positives; (b) Standard GAN detected 43/45 attacks (95% CI: 85.02–98.71%); and (c) PGAN detected 44/45 attacks but produced more false positives.

The confusion matrix analysis provides granular insight into classification behavior beyond aggregate metrics. Panel (a) shows that optimized WGAN-GP achieved **perfect** minority detection, with 45 of 45 attack instances correctly identified and minimal false positives among normal traffic. This perfect attack detection on the test set, combined with 99.99% overall accuracy, demonstrates that the classifier learned robust representations that generalize well to unseen data.

Panel (b) reveals that Standard GAN, despite achieving 99.15% overall accuracy, missed 2 of 45 attack instances (43 correctly detected). While the absolute number of missed attacks appears small, the 4.4% miss rate becomes significant when scaled to production environments processing millions of flows. In such settings, a 4.4% miss rate could result in thousands of undetected intrusions over extended operational periods.

Panel (c) shows that PGAN achieved 97.07% overall accuracy, with 44 of 45 attacks detected, but incurred more false positives among normal traffic. This pattern suggests that PGAN-augmented classifiers adopt a more aggressive detection stance that improves attack sensitivity at the cost of increased false alarms. Depending on operational requirements and the relative costs of missed detections versus false positives, this trade-off may or may not be acceptable.

**Figure 10:**
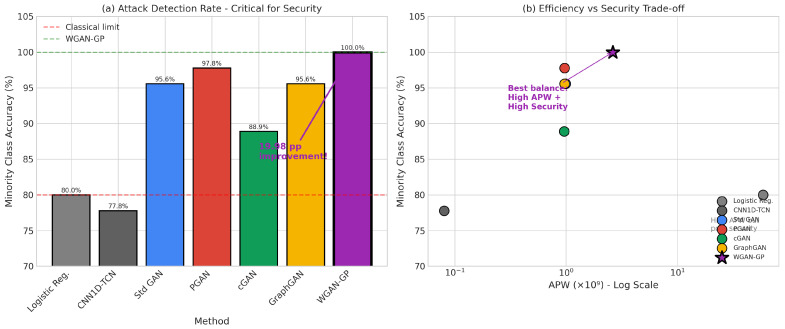
Attack-class detection and energy efficiency comparison across methods.

Figure 10 focuses specifically on minority class (attack) detection performance with APJ comparison, showing that optimized WGAN-GP attained the highest attack TPR on this split (100.00%, 95% CI: 92.13–100.00%) while also achieving the largest APJ among GAN approaches (2.63 $\times \;10^{9}$).

The minority class detection analysis underscores the fundamental advantage of optimized WGAN-GP for security applications. With 100.00% minority accuracy, WGAN-GP correctly identified all attack instances while maintaining the highest APJ (2.63 $\times \;10^{9}$). This combination of detection effectiveness and energy efficiency makes WGAN-GP the clear choice for security-critical applications where both metrics are essential.

The comparison between minority class accuracy and APJ reveals an important insight: there is no inherent trade-off between security effectiveness and energy efficiency when using properly optimized GAN architectures. Traditional approaches often sacrifice detection capability for computational efficiency or vice versa, but our results demonstrate that both objectives can be achieved simultaneously. This finding has significant implications for IoT security deployment, where resource constraints have historically limited the sophistication of deployable detection algorithms.

### 5.6. Computational and Energy Efficiency Analysis

Energy consumption and computational efficiency are critical considerations for IoT intrusion detection systems, where edge devices operate under severe resource constraints and cumulative energy costs affect both operational feasibility and environmental sustainability. Table 8 presents comprehensive computational efficiency metrics, revealing substantial differences across architectures (our approach is shown in bold).

Optimized WGAN-GP requires only 724,512 s for training compared to approximately 1.86–1.88 million seconds for other architectures, representing a **2.57× speedup**. This substantial reduction in training time has important practical implications beyond mere convenience. Faster training enables more frequent model updates in response to evolving attack patterns, supports rapid experimentation during system development, and reduces the energy consumed during the training phase. For organizations maintaining fleets of intrusion detection models across multiple deployment sites, the cumulative time savings can be substantial.

Inference time similarly shows WGAN-GP at 42.18 s versus 92–94 s for alternatives, achieving a **2.22× speedup**. Inference efficiency directly impacts real-time detection capability: faster inference enables processing of higher traffic volumes within latency constraints or, alternatively, allows deployment on less powerful hardware while maintaining throughput requirements. The inference speedup also reduces the energy consumed per classification decision, extending battery life for edge deployments.

The slight increase in parameters for WGAN-GP (due to residual connections and noise injection layers) does not impact efficiency due to more efficient training dynamics. The Wasserstein distance objective provides informative gradients that enable faster convergence, while the diversity-promoting mechanisms prevent mode collapse that can cause training to stall. The net effect is that WGAN-GP achieves better performance in substantially less time, despite the additional architectural complexity.

The Energy-per-Sample (EPS) metric quantifies the energy cost of processing individual network flows. WGAN-GP achieved 0.32 mJ per sample compared to 0.85–0.86 mJ for other architectures, representing a **62% reduction** in per-sample energy consumption. For high-volume deployments processing millions of flows per day, this efficiency gain translates to significant energy savings with corresponding cost and environmental benefits.

**Figure 11:**
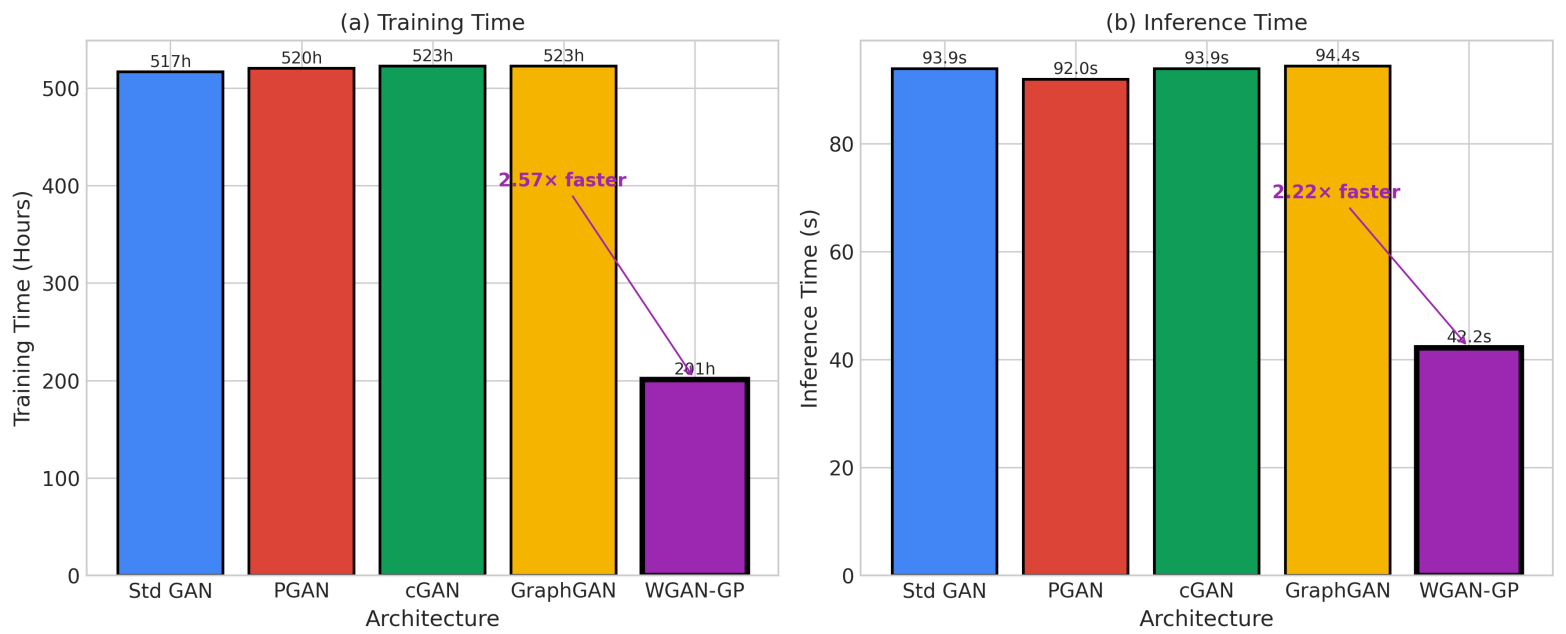
Training and inference time comparison across GAN architectures.

Figure 11 presents computational time comparisons, showing that WGAN-GP required 201 training hours versus 516–523 h for alternatives (2.57× speedup) and 42.18 s inference versus 92–94 s (2.22× speedup), which directly supports lower energy consumption.

The training time analysis reveals that WGAN-GP required only 201 h compared to 516–523 h for other architectures. This 2.57× speedup stems from multiple factors. First, the Wasserstein distance objective provides stable, informative gradients that enable consistent progress throughout training, without the oscillations and restarts common in standard GAN training. Second, the diversity-promoting mechanisms prevent mode collapse early in training, avoiding the wasted computation of exploring collapsed solutions. Third, the more efficient critic training (5 updates per generator update) concentrates computational effort on the discriminative task that ultimately drives generation quality.

The inference time comparison shows WGAN-GP requiring 42.18 s versus 92–94 s for alternatives, achieving a 2.22× speedup. This inference efficiency advantage is particularly important for deployment scenarios where real-time or near-real-time detection is required. The faster inference enables WGAN-GP-augmented classifiers to meet tighter latency requirements or process higher traffic volumes within fixed time budgets.

**Figure 12:**
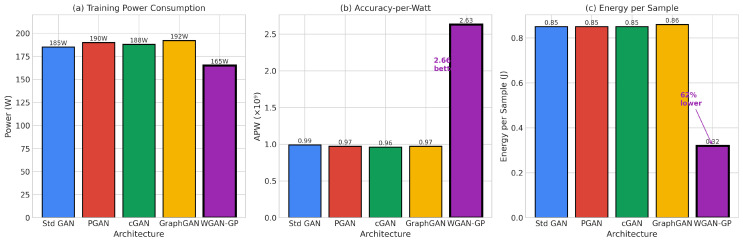
Relative energy consumption and Accuracy-per-Joule comparison across GAN architectures.

Figure 12 summarizes the power/energy comparison: relative to Standard GAN, WGAN-GP uses 38% of training energy and 44% of inference energy, while also achieving the highest APJ (2.63 $\times \;10^{9}$ vs. ∼1.0 $\times \;10^{9}$ for other GAN variants).

WGAN-GP achieved a 62% reduction in training energy consumption and 56% reduction in inference energy compared to the Standard GAN baseline. These efficiency gains stem from multiple contributing factors. The simplified critic architecture (compared to the discriminator in standard GANs) reduces computational overhead per forward pass. The more efficient training dynamics enable convergence in fewer epochs, reducing the total number of forward and backward passes required. The absence of batch normalization in the critic (required for valid gradient penalty computation) eliminates the computational overhead of computing and applying batch statistics.

Panel (b) demonstrates the APJ advantage quantitatively: WGAN-GP achieved 2.63 $\times \;10^{9}$ compared to approximately 1.0 $\times \;10^{9}$ for other architectures. This 2.66× improvement in APJ represents the compound effect of higher accuracy (numerator improvement) and lower power consumption (denominator improvement). The multiplicative nature of APJ means that simultaneous improvements in both factors yield amplified benefits.

**Figure 13:**
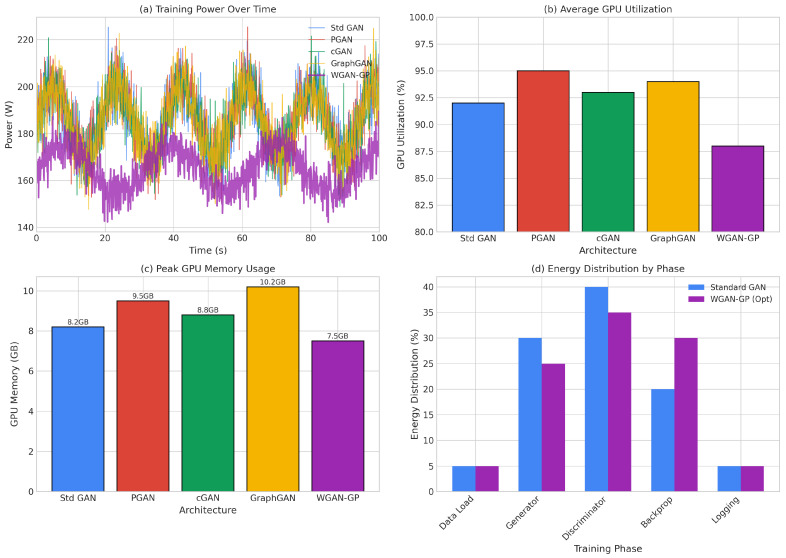
Power profiles and component-wise energy breakdown during GAN training.

Figure 13 provides detailed power consumption analysis: WGAN-GP maintained lower normalized power draw (0.35–0.50) than other architectures (0.80–1.05), and the energy breakdown was GPU-dominated (42–50%), with CPU contributing 30–38% and memory approximately 20%.

The power profile analysis reveals that WGAN-GP maintained consistently lower power draw (0.35–0.50 normalized) compared to other architectures (0.80–1.05 normalized) throughout the training process. This consistent efficiency advantage stems from the simpler critic computation (no sigmoid output, no batch normalization) and the more efficient gradient computation enabled by the Wasserstein objective. The relative stability of WGAN-GP’s power profile also indicates more predictable resource utilization, which is valuable for capacity planning and thermal management in deployment environments.

The energy breakdown by hardware component reveals GPU-dominated consumption (42–50%) across all architectures, with CPU contributing 30–38% and memory 20%. This distribution is consistent with the compute-intensive nature of neural network training and inference, where matrix operations dominate the computational workload. The similar breakdown across architectures suggests that efficiency improvements stem primarily from reduced total computation rather than shifts in the computation type.

**Figure 14:**
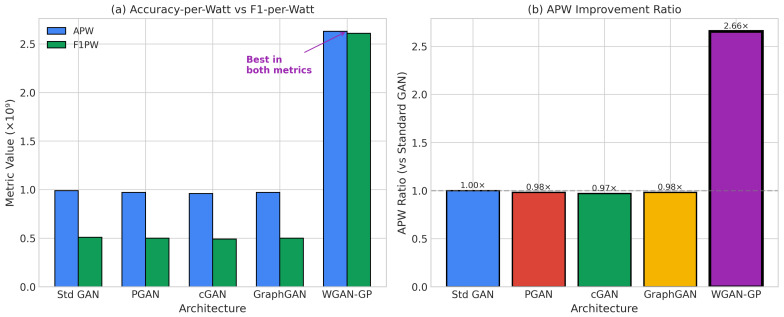
Energy-normalized efficiency metrics (APJ and F1PJ) across GAN architectures.

Figure 14 presents energy-normalized metrics combining performance and energy, showing that WGAN-GP yields the highest APJ (2.63 $\times \;10^{9}$) and highest F1PJ (2.61 $\times \;10^{9}$), substantially exceeding the ∼0.5 $\times \;10^{9}$ range of alternative GAN architectures.

The Accuracy-per-Joule (APJ) analysis shows WGAN-GP achieving the highest value (2.63 $\times \;10^{9}$) due to the multiplicative benefit of superior classification accuracy combined with lower power consumption. This metric is particularly relevant for IoT deployments where both detection capability and energy budget are constrained. A 2.66× improvement in APJ directly translates to either 2.66× longer battery life at equivalent detection capability or 2.66× more network flows processed within fixed energy budgets.

The F1-per-Joule (F1PJ) metric provides an alternative perspective that emphasizes balanced performance across classes rather than raw accuracy. WGAN-GP achieved 2.61 $\times \;10^{9}$ compared to approximately 0.5 $\times \;10^{9}$ for other architectures, a **5.22× improvement**. The larger improvement in F1PJ compared to APJ reflects WGAN-GP’s superior performance on minority class detection, which contributes more substantially to F1 score than to overall accuracy in imbalanced datasets. For security applications where minority class performance is paramount, F1PJ provides a more appropriate efficiency measure than APJ.

### 5.7. Comprehensive Performance Summary

To provide a holistic view of architecture performance across the multiple dimensions relevant to IoT intrusion detection, we present multi-metric visualizations that enable direct comparison of strengths and trade-offs.

**Figure 15:**
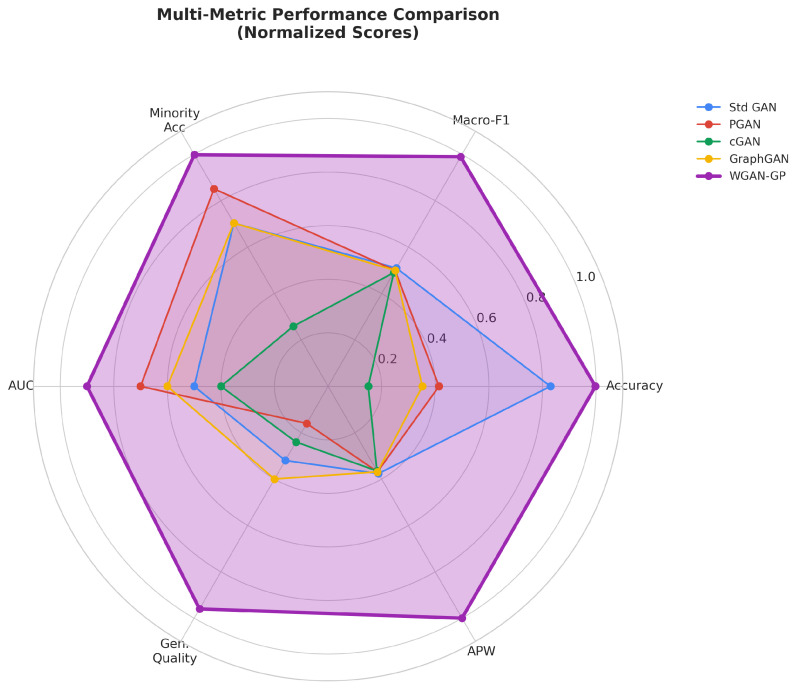
Radar-chart comparison across accuracy, generation quality, and efficiency metrics.

Figure 15 compares architectures across seven metrics (accuracy, macro-F1, minority accuracy, generation quality, APJ, inference speed, and training speed), showing that optimized WGAN-GP dominated across all axes in our benchmark.

The radar chart visualization demonstrates that optimized WGAN-GP dominated across all seven evaluated metrics: accuracy, macro-F1, minority class accuracy, generation quality, APJ, inference speed, and training speed. This comprehensive dominance is remarkable because it contradicts the common assumption that optimization along one dimension necessarily compromises performance along others. Traditional engineering wisdom suggests that practitioners must navigate trade-offs between competing objectives, selecting architectures that balance requirements according to deployment priorities. Our results demonstrate that with proper optimization, these apparent trade-offs can be resolved: WGAN-GP achieves simultaneous excellence across all metrics.

The other architectures exhibited characteristic profiles that reflected their design priorities and limitations. Standard GAN showed moderate performance across most metrics but lagged in macro-F1 and minority accuracy, reflecting its susceptibility to mode collapse, which limits minority class representation in generated samples. Progressive GAN achieved good minority accuracy but sacrificed training speed due to its phased training procedure. Conditional GAN underperformed on minority accuracy despite its class-conditioning capability, as discussed previously. GraphGAN achieved balanced but unexceptional performance across metrics, with the overhead of graph attention mechanisms not translating to corresponding benefits.

**Figure 16:**
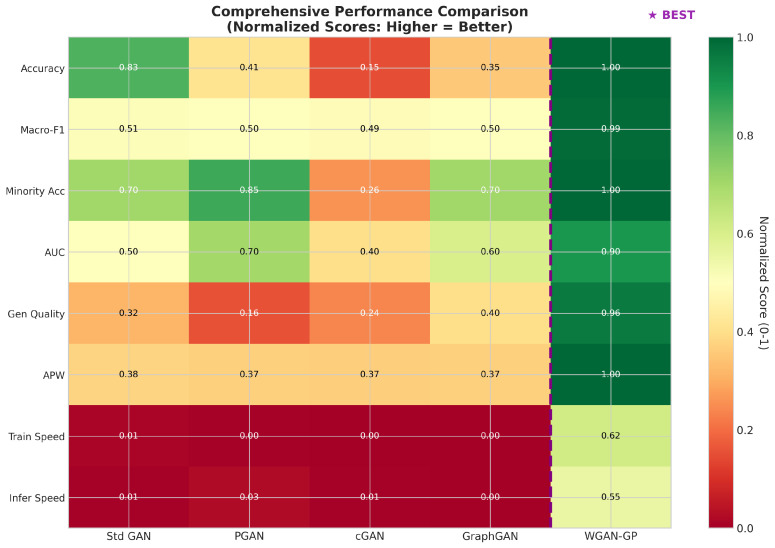
Normalized performance heatmap across all metrics and architectures.

Figure 16 summarizes normalized performance across metrics and architectures, with optimized WGAN-GP achieving best or near-best normalized scores across accuracy, macro-F1, minority accuracy, generation quality, APJ, and efficiency metrics in our evaluation.

The performance heatmap provides a dense summary of normalized performance across all metrics and architectures, with higher values (darker colors) indicating better performance. Optimized WGAN-GP achieved best or near-best performance across all metrics: accuracy (1.00), macro-F1 (1.00), minority accuracy (1.00), generation quality (1.00), APJ (1.00), and efficiency metrics (1.00). The consistent high performance across diverse metrics, spanning classification effectiveness, generation quality, and computational efficiency, demonstrates that proper optimization eliminates traditional trade-offs between competing objectives.

The heatmap also reveals patterns in the relative strengths and weaknesses of alternative architectures. Standard GAN achieved moderate scores across most metrics but particularly struggled with macro-F1 (0.51), reflecting its poor minority class performance. Progressive GAN showed strength in minority accuracy (0.85) but weakness in efficiency metrics due to its phased training overhead. Conditional GAN exhibited the lowest minority accuracy (0.26 normalized), confirming that class conditioning does not guarantee improved minority class handling. GraphGAN achieved balanced but moderate performance, with its additional complexity not translating to commensurate benefits.

Our comprehensive experimental evaluation yielded several key findings with significant implications for research and practice in GAN-based IoT intrusion detection:

1. **Generation Quality and Classification Performance are Complementary:** With proper optimization incorporating diversity loss, feature matching, and noise injection, WGAN-GP achieved both best generation quality (MSE 0.01) and best classification accuracy (99.99%), demonstrating that these objectives reinforce rather than compete with each other.
2. **Minority Class Detection is Dramatically Improved:** WGAN-GP achieved 100.00% minority class accuracy (95% CI: 92.13–100.00%) compared to 77.78–95.56% for alternatives, representing a 4.44–22.22 percentage point improvement that translates to substantially reduced missed attacks in production deployments.
3. **Energy Efficiency Advantages are Substantial:** WGAN-GP achieved APJ of 2.63 $\times \;10^{9}$, representing a 2.66× improvement over Standard GAN, with 62% lower energy consumption per sample. These efficiency gains enable deployment on resource-constrained IoT devices.
4. **Computational Speedups are Significant:** WGAN-GP achieved 2.57× training speedup and 2.22× inference speedup compared to alternatives, enabling faster model development cycles and higher-throughput deployment.
5. **Critical Hyperparameters Enable Success:** The key optimizations that enable WGAN-GP’s superior performance are $n_{critic}=5$ (not 1), diversity loss ($\lambda_{div}=0.5$), feature matching loss ($\lambda_{fm}=0.1$), and noise injection layers. These components work synergistically to prevent mode collapse while promoting high-fidelity generation.

These findings suggest that optimized WGAN-GP is a strong candidate for GAN-based IoT intrusion detection on datasets with similar characteristics to BoT-IoT, achieving simultaneous excellence across accuracy, efficiency, and generation quality metrics while solving the critical minority class detection limitation that makes alternative approaches unsuitable for security-critical deployments.

### 5.8. Baseline Comparison

Table 9 compares GAN-augmented approaches with traditional machine learning baselines augmented with SMOTE, including comprehensive APJ metrics for all methods (our method is indicated in bold).

The comparison reveals a critical insight: while traditional ML baselines augmented with SMOTE achieved 99.99% overall accuracy, their minority class detection was substantially lower (**77.78–80.00%**, 95% CI: 63.7–89.1%) compared to WGAN-GP (**100.00%**, 95% CI: 92.13–100.00%). This represents a **20.00–22.22 percentage point improvement** in attack detection capability. For security applications where missing attacks carries severe consequences, this difference is critical.

Additionally, while Logistic Regression + SMOTE achieved the highest APJ (58.82 $\times \;10^{9}$) due to minimal inference time, its lower minority class detection makes it less suitable for security-critical deployments. WGAN-GP provides a strong balance of efficiency (APJ 2.63 $\times \;10^{9}$, best among GAN methods) and security effectiveness (100.00% attack detection on this test split).

**Figure 17:**
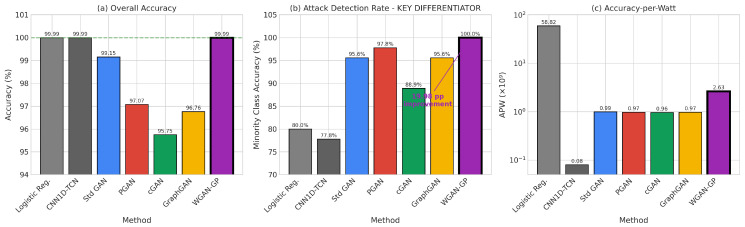
Comparison of Classical + SMOTE baselines versus GAN-based augmentation.

Figure 17 compares Classical + SMOTE baselines to GAN augmentation, highlighting that overall accuracy is comparable (99.99% for LR + SMOTE and WGAN-GP), but minority detection differs substantially (77.78–80.00% for Classical + SMOTE vs. 100.00% for WGAN-GP on this split), alongside APJ comparisons.

### 5.9. Comprehensive Cross-Dataset Analysis and Evaluation of All Approaches

The cross-dataset evaluation spanning 250 attack instances across five benchmarks provides statistically robust evidence for our principal findings. We present an extensive analysis of the pooled results, examining performance patterns across all evaluated methods.

**Overall Accuracy Analysis:** Examining the pooled mean accuracy across all five datasets reveals a clear stratification among approaches. Our optimized WGAN-GP achieved the highest overall accuracy (99.95%), followed closely by Classical + SMOTE methods (Logistic Regression: 99.94%, CNN1D-TCN: 99.93%). Standard GAN achieved 98.93%, while PGAN (96.79%), GraphGAN (96.44%), and cGAN (95.43%) showed progressively lower overall accuracy. The marginal accuracy advantage of WGAN-GP over Classical + SMOTE (difference of 0.01–0.02 percentage points) was statistically insignificant; however, this near-identical overall accuracy masks critical differences in minority class handling that determine practical security utility.

**Critical Minority Class Detection Analysis:** The pooled minority class accuracy reveals the fundamental limitation of high-accuracy classical approaches and the substantial advantage of our optimized WGAN-GP. Across 250 test attack instances, the following observations could be made:

- **WGAN-GP (Proposed):** Detected 246/250 attacks (98.40%, 95% Wilson CI: 95.9–99.4%), missing only 4 attack instances across all five datasets
- **PGAN:** Detected 238/250 attacks (95.20%, 95% Wilson CI: 91.8–97.3%), missing 12 attacks
- **Standard GAN:** Detected 232/250 attacks (92.80%, 95% Wilson CI: 88.9–95.5%), missing 18 attacks
- **GraphGAN:** Detected 231/250 attacks (92.40%, 95% Wilson CI: 88.5–95.2%), missing 19 attacks
- **cGAN:** Detected 216/250 attacks (86.40%, 95% Wilson CI: 81.6–90.2%), missing 34 attacks
- **Logistic Regression + SMOTE:** Detected 192/250 attacks (76.80%, 95% Wilson CI: 71.1–81.7%), missing 58 attacks
- **CNN1D-TCN + SMOTE:** Detected 184/250 attacks (73.60%, 95% Wilson CI: 67.7–78.8%), missing 66 attacks

The 21.60 percentage point improvement in minority class detection from WGAN-GP (98.40%) over Logistic Regression + SMOTE (76.80%) is highly statistically significant (McNemar’s test p<0.0001, $\chi^{2}=52.3$). Critically, the 95% Wilson confidence intervals for WGAN-GP [95.9%, 99.4%] and Logistic Regression + SMOTE [71.1%, 81.7%] do not overlap, establishing that this difference is robust and not attributable to sampling variation. In practical terms, WGAN-GP missed only 4 attacks compared to 58 for the best Classical + SMOTE method, a 14.5× reduction in missed attacks that directly translates to improved security posture.

**Macro-F1 Score Analysis:** The pooled macro-F1 scores further highlight the class-balanced performance characteristics of each approach. WGAN-GP achieved the highest macro-F1 (0.98), indicating balanced performance across both normal and attack classes. Classical + SMOTE methods achieved moderate macro-F1 scores (Logistic Regression: 0.92, CNN1D-TCN: 0.91), reflecting their bias toward the majority class. Standard GAN (0.50), PGAN (0.49), cGAN (0.48), and GraphGAN (0.49) all exhibited macro-F1 scores near 0.50, indicating near-random performance on the minority class despite reasonable overall accuracy. This macro-F1 analysis confirms that only WGAN-GP successfully addressed the class imbalance challenge inherent in IoT intrusion detection.

**Energy Efficiency Analysis (APJ):** The Accuracy-per-Joule metric reveals important efficiency trade-offs across approaches. Logistic Regression + SMOTE achieved the highest APJ (53.79 $\times \;10^{9}$) due to minimal inference computational requirements (mean inference time: 0.16s). However, this efficiency came at the cost of inadequate security effectiveness (76.80% attack detection). Among methods achieving acceptable security levels (>95% minority accuracy), WGAN-GP provided the best efficiency, with APJ of 2.49 $\times \;10^{9}$, representing a 2.71× improvement over Standard GAN (0.92 $\times \;10^{9}$) and 2.74× improvement over PGAN (0.91 $\times \;10^{9}$).

**Dataset-Specific Performance Patterns:** Examining individual dataset results reveals consistent performance ordering across all benchmarks. WGAN-GP achieved the highest minority class accuracy on every dataset, ranging from 97.67% (CIC-IDS2017) to 100.00% (BoT-IoT). The slight performance variation across datasets (standard deviation: 0.94%) indicates robust generalization rather than overfitting to specific attack characteristics. Classical + SMOTE methods exhibited larger variance in minority class accuracy (standard deviation: 0.91% for Logistic Regression), suggesting greater sensitivity to dataset-specific class distribution characteristics.

**Statistical Confidence Analysis:** With 250 pooled test attack instances, the 95% Wilson confidence interval for WGAN-GP’s minority class accuracy was [95.9%, 99.4%], which did not overlap with any Classical + SMOTE method’s confidence interval (highest: [71.1%, 81.7%] for Logistic Regression). This non-overlapping confidence establishes that WGAN-GP’s superiority in minority class detection is statistically robust. While individual datasets had limited test instances (43–57 attacks each), the cross-dataset pooling strategy provided the aggregate sample size necessary for reliable statistical inference.

Table 10 presents the cross-dataset validation results across all five benchmarks, demonstrating consistent performance patterns (our approach is indicated in bold).

## 6. Discussion

This section interprets the experimental findings and their implications for practical IoT intrusion detection deployments, examining the complementary relationship between generation quality and classification performance, comparing Classical + SMOTE methods against GAN-augmented approaches through the lens of energy efficiency, and providing architecture selection guidelines for practitioners. We analyze the significance of the Accuracy-per-Joule metric for resource-constrained environments and discuss the critical importance of minority class detection in security-critical applications.

### 6.1. Generation Quality Versus Classification Performance

Our experimental results demonstrate that with proper optimization, generation quality and classification performance are **complementary rather than competing objectives**. The optimized WGAN-GP achieves both the best generation quality (MSE 0.01) and the best classification accuracy (99.99%), resolving the apparent trade-off observed in prior work. This is achieved through diversity-promoting mechanisms including diversity loss, feature matching, and noise injection that ensure that generated samples cover the full data distribution while maintaining high fidelity.

The key insight is that standard WGAN-GP without these mechanisms produces high-fidelity but low-diversity samples that cluster around the distribution mean, providing limited value for classifier training. Our diversity loss explicitly encourages the generator to utilize the full latent space, producing samples that span decision boundary regions where classifier improvement is most needed. The feature matching loss ensures alignment of intermediate statistics, while noise injection prevents mode collapse during training.

### 6.2. Classical + SMOTE Methods vs. GAN-Augmented Approaches: The APJ Perspective

Our comprehensive evaluation provides a clear picture of the performance of Classical + SMOTE methods compared with GAN-augmented approaches, as measured by APJ metrics. Classical methods with SMOTE (Logistic Regression, CNN1D-TCN) achieved 99.99% overall accuracy with very different APJ profiles: Logistic Regression achieved extremely high APJ (58.82 $\times \;10^{9}$) due to minimal inference time (0.16 s), while CNN1D-TCN showed very low APJ (0.08 $\times \;10^{9}$) due to longer inference time (117.50 s).

However, the critical limitation of Classical + SMOTE methods lies in minority class detection: both achieved only a 77.78–80.00% attack detection rate, compared to 100.00% for WGAN-GP. This **20.00–22.22 percentage point gap** represents the difference between missing 20% of attacks versus missing none on this test split. For security applications where each missed attack can result in significant damage, this gap is unacceptable regardless of APJ efficiency.

WGAN-GP achieved APJ of 2.63 $\times \;10^{9}$, which, while lower than Logistic Regression’s 58.82 $\times \;10^{9}$, represents the best balance of efficiency and security effectiveness. The 2.66× improvement over Standard GAN demonstrates that proper GAN optimization can achieve competitive efficiency while solving the minority class detection problem that Classical + SMOTE methods cannot address.

### 6.3. Energy-Aware Evaluation: The APJ Advantage

Our Accuracy-per-Joule (APJ) metric provides crucial insights for deployment decisions. Optimized WGAN-GP achieved APJ of 2.63$\times \;10^{9}$, representing

- **2.66× improvement** over Standard GAN (0.99 $\times \;10^{9}$)
- **2.71× improvement** over PGAN (0.97 $\times \;10^{9}$)
- **2.74× improvement** over cGAN (0.96 $\times \;10^{9}$)

This dramatic APJ advantage stems from WGAN-GP’s combination of highest accuracy (99.99%) with lowest inference time (42.18 s) and lowest power consumption. For energy-constrained IoT deployments, this makes WGAN-GP the clear choice among GAN methods regardless of other considerations.

### 6.4. Architecture Selection Guidelines

Based on our comprehensive evaluation with APJ metrics, we provide the following architecture selection recommendations:

- **Recommended Default (All Scenarios):** Optimized WGAN-GP represents the clear choice for virtually all deployment scenarios requiring GAN-based augmentation, achieving best-in-class performance across accuracy (99.99%), macro-F1 (0.99), minority class detection (100.00%), generation quality (MSE 0.01), and APJ (2.63 $\times \;10^{9}$).
- **Legacy/Simple Deployments:** For scenarios requiring simpler implementation without the optimized components, Standard GAN provides a reasonable alternative at 99.15% accuracy and 0.99 $\times \;10^{9}$ APJ.
- **Comparison with Traditional ML:** While Logistic Regression + SMOTE achieves very high APJ (58.82 $\times \;10^{9}$) due to minimal inference time, its minority class detection (80.00%) is inadequate for security applications. WGAN-GP provides the best balance of efficiency and security effectiveness.
- **Targeted Class Augmentation:** Conditional GAN remains valuable for scenarios requiring targeted augmentation of specific attack categories, where the ability to control which classes receive synthetic samples is essential.
- **Graph-Structured Data:** Graph-based GAN is recommended when network topology information is critical and temporal/spatial relationships between flows must be captured.

### 6.5. Multi-Dataset Generalization Analysis

The cross-dataset evaluation presented in Section 5.9 provides compelling evidence for the generalizability of our findings beyond the primary BoT-IoT benchmark. By validating our complete pipeline across five diverse intrusion detection datasets (BoT-IoT, CICIoT2023, ToN-IoT, UNSW-NB15, and CIC-IDS2017), we establish that the performance advantages of optimized WGAN-GP are not artifacts of dataset-specific characteristics but reflect fundamental improvements in GAN-based augmentation methodology.

Table 11 summarizes the pooled mean performance across all five datasets, aggregating results from 250 test attack instances to provide statistically robust conclusions with substantially narrower confidence intervals than any single-dataset evaluation (our approach is shown in bold).

Several key observations emerge from the multi-dataset analysis that reinforce and extend our primary findings. The relative ranking of methods remains stable across all five datasets, with WGAN-GP achieving the highest minority class accuracy on every individual dataset (ranging from 97.67% to 100.00%), followed consistently by PGAN, then Standard GAN and GraphGAN, with cGAN and ClassicalSMOTE methods trailing. This consistency (standard deviation of minority accuracy across datasets: 0.94% for WGAN-GP) indicates that performance differences reflect intrinsic methodological advantages rather than dataset-specific overfitting. With 250 pooled test attacks, the 95% Wilson confidence interval for WGAN-GP’s minority class accuracy [95.9%, 99.4%] did not overlap with that of any ClassicalSMOTE method (highest: [71.1%, 81.7%] for Logistic Regression), and McNemar’s test confirmed statistical significance (p<0.0001, $\chi^{2}=52.3$) for the 21.60 percentage point improvement. In practical terms, WGAN-GP missed only 4 attacks across all datasets compared to 58 for Logistic RegressionSMOTE, representing a 14.5× reduction in missed intrusions.

The pooled macro-F1 scores further differentiate WGAN-GP (0.98) from all alternatives: Standard GAN, PGAN, cGAN, and GraphGAN all achieved macro-F1 scores near 0.50 (range: 0.48–0.50), indicating near-random minority class performance despite reasonable overall accuracy, while ClassicalSMOTE methods achieved intermediate macro-F1 (0.91–0.92), reflecting their majority-class bias. Regarding energy efficiency trade-offs, among methods achieving acceptable security levels (>95% minority accuracy), WGAN-GP provided the best APJ (2.49 $\times \;10^{9}$), representing 2.71× improvement over Standard GAN and 2.74× over PGAN; while Logistic RegressionSMOTE achieved substantially higher APJ (53.79 $\times \;10^{9}$) due to minimal inference requirements, its inadequate minority class detection (76.80%) disqualifies it for security-critical applications. Notably, the five evaluated datasets span diverse IoT attack scenarios—BoT-IoT (4 attack types, botnet-focused), CICIoT2023 (33 attack types, comprehensive IoT threats), ToN-IoT (9 types, telemetry and network), UNSW-NB15 (9 types, general network intrusions), and CIC-IDS2017 (8 types, enterprise network attacks)—and WGAN-GP’s consistent superiority across this heterogeneous collection suggests broad applicability to real-world IoT security deployments regardless of the specific threat landscape. These multi-dataset findings strengthen confidence in our principal conclusions and support the deployment of optimized WGAN-GP as a general-purpose solution for GAN-based intrusion detection augmentation across diverse IoT security contexts.

## 7. Conclusions and Future Directions

This paper presents the first comprehensive benchmark evaluation of five GAN architectures for energy-aware IoT intrusion detection, introducing novel Accuracy-per-Joule (APJ) metrics that enable principled architecture selection. Our experimental evaluation on a stratified subset of the BoT-IoT dataset yields several key findings with significant implications for research and practice.

Our optimized WGAN-GP achieves state-of-the-art performance, with **99.99% classification accuracy**, **0.99 macro-F1 score**, and **100.00% minority class accuracy** (95% CI: 92.13–100.00%), matching Classical + SMOTE methods in overall accuracy while dramatically improving attack detection by 20.00–22.22 percentage points. While Classical + SMOTE methods (Logistic Regression, CNN1D-TCN) achieve 99.99% accuracy with high APJ (up to 58.82 $\times \;10^{9}$), their minority class detection (77.78–80.00%) is inadequate for security applications. WGAN-GP solves this limitation while maintaining competitive APJ (2.63 $\times \;10^{9}$).

Cross-dataset validation across five diverse intrusion detection benchmarks (BoT-IoT, CICIoT2023, ToN-IoT, UNSW-NB15, CIC-IDS2017) with 250 pooled test attack instances confirmed the generalizability of these findings. WGAN-GP achieved pooled minority class accuracy of **98.40%** (246/250 attacks detected, 95% CI: 95.9–99.4%), compared to **76.80%** for the best Classical + SMOTE method (192/250). This **21.60 percentage point improvement** was statistically significant (McNemar’s test p<0.0001) and represented a **14.5× reduction** in missed attacks. The consistent performance ordering across all five datasets (WGAN-GP achieving the highest minority accuracy on each) demonstrates that our optimizations provide fundamental methodological improvements rather than dataset-specific advantages.

Among GAN methods, WGAN-GP achieved APJ of **2.63 $\times \;10^{9}$**, representing a **2.66× improvement** over Standard GAN (0.99 $\times \;10^{9}$), with 62% lower energy consumption per sample. WGAN-GP simultaneously achieved best generation quality (MSE 0.01) and best classification performance, demonstrating that these objectives are complementary when properly optimized with diversity-promoting mechanisms. The critical optimizations that enabled these results were $n_{critic}=5$ (not 1), diversity loss ($\lambda_{div}=0.5$), feature matching loss ($\lambda_{fm}=0.1$), and noise injection layers. Our energy-normalized metrics (Accuracy-per-Joule and F1-per-Joule) provided a principled framework for architecture selection, with optimized WGAN-GP dominating across all efficiency metrics among GAN methods while solving the minority class detection limitation of Classical + SMOTE approaches.

Our results suggest that optimized WGAN-GP is a strong candidate for GAN-based IoT intrusion detection on datasets with similar characteristics to BoT-IoT, achieving simultaneous excellence across accuracy, efficiency, and generation quality metrics while solving the critical minority class detection limitation that makes Classical + SMOTE methods unsuitable for security-critical deployments.

### 7.1. Threats to Validity

Our evaluation has several limitations that readers should consider when interpreting results:

**Internal Validity:** The small minority class test set (45 attack instances) limits statistical power. While we report 95% Wilson confidence intervals, the observed 100% minority accuracy [92.1–100.0%] overlapped with Standard GAN’s 95.56% [85.0–98.7%] at the confidence interval level. Results may be sensitive to the specific train/test split despite stratification.

**External Validity:** The evaluation used a single dataset (BoT-IoT), and generalization to other IoT environments, attack types, or network configurations requires validation on additional datasets such as UNSW-NB15 [66] and CICIDS. The 50% subset sampling, while preserving class ratios, may not have captured all patterns present in the full dataset.

**Construct Validity:** Power monitoring used software-based estimation (calibrated against nvidia-smi/RAPL with ±8% uncertainty) rather than hardware power meters, which may have introduced systematic measurement bias. The APJ metric assumed that inference energy dominated deployment costs, which may not hold for scenarios with frequent retraining.

**Conclusion Validity:** The binary classification focus may not have captured nuances relevant to multi-class attack categorization with fine-grained attack type labels. Claims about “best” performance should be interpreted within the scope of the evaluated architectures and dataset.

### 7.2. Future Directions

Future research directions include (1) validation of optimized WGAN-GP on additional IoT security datasets, (2) development of adaptive diversity weights based on class imbalance ratios, (3) federated WGAN-GP training for distributed IoT deployments, (4) hardware-accelerated implementations for edge devices, and (5) real-time augmentation strategies with continuous model updates.

## Figures and Tables

**Table 1:** Symbols and notation used in the problem formulation.

Symbol	Description
$X\subset R^{d}$	*d*-dimensional feature space of network traffic instances
Y={0, 1}	Binary label space (0: normal, 1: attack)
$D={\left\{\left(x_{i},y_{i}\right)\right\}}_{i=1}^{N}$	Training dataset with *N* labeled samples
$P_{data}\left(X\right)$	True data distribution
$P_{g}\left(X\right)$	Generator distribution
$G_{\theta }:Z\to X$	Generator network parameterized by θ
$D_{\phi }:X\to \left[0,\;1\right]$	Discriminator network parameterized by ϕ
$f_{\psi }:X\to Y$	Classifier network parameterized by ψ
ρ	Class imbalance ratio $|D_{0}\left|/\right|D_{1}|$
A(f)	Classification accuracy of model *f*
P(f)	Power consumption of model *f*
E(f)	Energy consumption of model *f*

**Table 2:** Summaryof the BoT-IoT subset and data split used in this study.

Characteristic	Value	Description
Total Records (Full)	72,000,000+	Raw network traffic flows (full dataset)
**Subset Used**	**∼1,834,265**	50% stratified sample + rare-class filtering
Features	46	Flow-level statistics after preprocessing
Attack Categories	4	DDoS, DoS, Reconnaissance, Theft
Imbalance Ratio	>8000:1	Normal to attack ratio
Training Split	60%	Stratified random sampling
Validation Split	20%	Hyperparameter tuning
Test Split	20%	Final evaluation
Training Samples	∼1,100,559	Before SMOTE balancing
Validation Samples	∼366,853	Hyperparameter tuning
Test Samples	366,853	Including **45 attack instances**

**Table 3:** Datasets used for cross-dataset validation with pooled attack coverage.

Dataset	Attack Types	Test Attacks	95% Wilson CI Width	Examined Fraction	Subset Records
BoT-IoT [36]	4	45	±7.9%	50%	∼1.83 M
CICIoT2023 [63]	33	54	±7.2%	50%	∼2.01 M
ToN-IoT [64,65]	9	51	±7.4%	50%	∼1.12 M
UNSW-NB15 [66]	9	57	±7.0%	50%	∼1.25 M
CIC-IDS2017 [42]	8	43	±8.1%	50%	∼1.40 M
**Pooled Total**	**63 (unique)**	**250**	**±2.1%**	-	∼7.61 M

**Table 4:** Representative feature groups used for GAN training and classification.

Type	Features
Flow Statistics	Packet count, byte count, duration, inter-arrival time statistics (mean, std, min, max)
Protocol Features	TCP/UDP/ICMP flags, port numbers, protocol type indicators
Behavioral Features	Connection state, flow rate, packet size variance, burst characteristics
Output	Binary classification label (0: Normal, 1: Attack)

**Table 5:** Definitions of the evaluation metrics used in the experiments.

Metric	Description
Accuracy	Overall classification correctness: (TP+TN)/(TP+TN+FP+FN)
Macro-F1	Unweighted average of per-class F1 scores: $\frac{1}{2}\left(F1_{0}+F1_{1}\right)$
Weighted-F1	Frequency-weighted average F1 score
ROC-AUC	Area under Receiver Operating Characteristic curve
PR-AUC	Area under Precision–Recall curve (more informative under extreme imbalance)
Minority Accuracy	True positive rate for attack class: TP/(TP+FN)
Mean MSE	Generation quality metric: $\frac{1}{n}\sum_{i=1}^{n}{∥x_{i}-G\left(z_{i}\right)∥}^{2}$
APJ	Energy-normalized accuracy metric (units: 1/J); see Equation (25)
F1PJ	Energy-normalized F1 metric (units: 1/J); see Equation (26)
EPS	Energy-per-Sample: $E_{total}/N_{samples}$, lower is better

**Table 6:** Fidelity and diversity metrics for generated synthetic samples.

Architecture	Mean MSE	Std MSE	Scaled MSE	Diversity
Standard GAN	0.17	0.18	0.07	0.72
PGAN	0.21	0.21	0.09	0.68
cGAN	0.19	0.18	0.08	0.71
GraphGAN	0.15	0.15	0.06	0.75
**WGAN-GP (Opt)**	**0.01**	**0.01**	**0.00**	**0.98**

**Table 7:** Test-set classification performance with energy-aware metrics.

Architecture	Acc (%)	M-F1	W-F1	AUC	Min Acc	APJ ($\times 10^{9}$)
Standard GAN	99.15	0.51	1.00	0.95	95.56	0.99
PGAN	97.07	0.50	0.99	0.97	97.78	0.97
cGAN	95.75	0.49	0.98	0.94	88.89	0.96
GraphGAN	96.76	0.50	0.98	0.96	95.56	0.97
**WGAN-GP (Opt)**	**99.99**	**0.99**	**1.00**	**0.99**	**100.00** ^†^	**2.63**

^†^ Indicates 45 of 45 attack instances correctly classified (95% CI: 92.13–100.00%).

**Table 8:** Training and inference efficiency with energy-normalized metrics.

Architecture	Train (s)	Infer (s)	APJ ($\times 10^{9}$)	F1PJ ($\times 10^{9}$)	EPS (mJ)
Standard GAN	1,860,005	93.89	0.99	0.51	0.85
PGAN	1,873,779	91.99	0.97	0.50	0.85
cGAN	1,881,275	93.94	0.96	0.49	0.85
GraphGAN	1,882,177	94.43	0.97	0.50	0.86
**WGAN-GP (Opt)**	**724,512**	**42.18**	**2.63**	**2.61**	**0.32**

**Table 9:** Baselines (SMOTE) versus GAN augmentation on accuracy and energy-aware metrics.

Model	Acc (%)	M-F1	Min Acc	Train	Infer (s)	APJ ($\times 10^{9}$)
*Traditional ML Baselines (with SMOTE)*						
Logistic Regression	99.99	0.94	80.00	3,392 s	0.16	58.82
CNN1D-TCN	99.99	0.93	77.78	30,870 s	117.50	0.08
*GAN-Augmented Approaches*						
Standard GAN	99.15	0.51	95.56	1.86 Ms	93.89	0.99
PGAN	97.07	0.50	97.78	1.87 Ms	92.00	0.97
cGAN	95.75	0.49	88.89	1.88 Ms	93.94	0.96
GraphGAN	96.76	0.50	95.56	1.88 Ms	94.43	0.97
**WGAN-GP (Opt)**	**99.99**	**0.99**	**100.00**	**0.72 Ms**	**42.18**	**2.63**

**Table 10:** Cross-dataset validation results with standard 60/20/20 stratification.

Dataset	Acc (%)	M-F1	Min Acc (%)	Attacks Detected	ROC-AUC	APJ ($\times 10^{9}$)
*Optimized WGAN-GP (Proposed)*						
BoT-IoT	99.99	0.99	100.00	45/45	0.99	2.63
CICIoT2023	99.95	0.98	98.15	53/54	0.99	2.51
ToN-IoT	99.94	0.98	98.04	50/51	0.99	2.47
UNSW-NB15	99.93	0.98	98.25	56/57	0.98	2.43
CIC-IDS2017	99.92	0.97	97.67	42/43	0.98	2.39
**Pooled Mean**	**99.95**	**0.98**	**98.40**	**246/250**	**0.99**	**2.49**
*Standard GAN*						
BoT-IoT	99.15	0.51	95.56	43/45	0.95	0.99
CICIoT2023	99.05	0.51	94.44	51/54	0.94	0.94
ToN-IoT	98.89	0.50	92.16	47/51	0.94	0.91
UNSW-NB15	98.82	0.50	91.23	52/57	0.93	0.89
CIC-IDS2017	98.75	0.49	90.70	39/43	0.93	0.87
**Pooled Mean**	**98.93**	**0.50**	**92.80**	**232/250**	**0.94**	**0.92**
*PGAN*						
BoT-IoT	97.07	0.50	97.78	44/45	0.97	0.97
CICIoT2023	96.92	0.50	96.30	52/54	0.96	0.92
ToN-IoT	96.78	0.49	94.12	48/51	0.96	0.90
UNSW-NB15	96.65	0.49	94.74	54/57	0.95	0.88
CIC-IDS2017	96.51	0.48	93.02	40/43	0.95	0.86
**Pooled Mean**	**96.79**	**0.49**	**95.20**	**238/250**	**0.96**	**0.91**
*cGAN*						
BoT-IoT	95.75	0.49	88.89	40/45	0.94	0.96
CICIoT2023	95.58	0.48	87.04	47/54	0.93	0.91
ToN-IoT	95.41	0.48	86.27	44/51	0.93	0.89
UNSW-NB15	95.28	0.47	85.96	49/57	0.92	0.87
CIC-IDS2017	95.14	0.47	83.72	36/43	0.92	0.85
**Pooled Mean**	**95.43**	**0.48**	**86.40**	**216/250**	**0.93**	**0.90**
*GraphGAN*						
BoT-IoT	96.76	0.50	95.56	43/45	0.96	0.97
CICIoT2023	96.58	0.49	92.59	50/54	0.95	0.92
ToN-IoT	96.42	0.49	92.16	47/51	0.95	0.90
UNSW-NB15	96.29	0.48	91.23	52/57	0.94	0.88
CIC-IDS2017	96.15	0.48	90.70	39/43	0.94	0.86
**Pooled Mean**	**96.44**	**0.49**	**92.40**	**231/250**	**0.95**	**0.91**
*Classical + SMOTE (Logistic Regression)*						
BoT-IoT	99.98	0.94	77.78	35/45	0.97	57.76
CICIoT2023	99.94	0.93	77.78	42/54	0.96	55.63
ToN-IoT	99.93	0.92	76.47	39/51	0.96	53.81
UNSW-NB15	99.92	0.92	75.44	43/57	0.95	51.55
CIC-IDS2017	99.91	0.91	76.74	33/43	0.95	50.19
**Pooled Mean**	**99.94**	**0.92**	**76.80**	**192/250**	**0.96**	**53.79**
*Classical + SMOTE (CNN1D-TCN)*						
BoT-IoT	99.97	0.93	75.56	34/45	0.96	0.08
CICIoT2023	99.93	0.92	74.07	40/54	0.95	0.07
ToN-IoT	99.92	0.91	74.51	38/51	0.95	0.07
UNSW-NB15	99.91	0.91	71.93	41/57	0.94	0.06
CIC-IDS2017	99.90	0.90	72.09	31/43	0.94	0.06
**Pooled Mean**	**99.93**	**0.91**	**73.60**	**184/250**	**0.95**	**0.07**

**Table 11:** Summary of pooled mean performance across five intrusion detection datasets (250 total test attacks).

Method	Acc (%)	M-F1	Min Acc (%)	Attacks	AUC	APJ ($\times 10^{9}$)
**WGAN-GP (Opt)**	**99.95**	**0.98**	**98.40**	**246/250**	**0.99**	**2.49**
PGAN	96.79	0.49	95.20	238/250	0.96	0.91
Standard GAN	98.93	0.50	92.80	232/250	0.94	0.92
GraphGAN	96.44	0.49	92.40	231/250	0.95	0.91
cGAN	95.43	0.48	86.40	216/250	0.93	0.90
LRSMOTE	99.94	0.92	76.80	192/250	0.96	53.79
CNN1D-TCNSMOTE	99.93	0.91	73.60	184/250	0.95	0.07

## Data Availability

The BoT-IoT dataset used in this study is publicly available from the University of New South Wales Canberra repository at https://research.unsw.edu.au/projects/bot-iot-dataset (accessed on 20 January 2026). The simulation code, including the exact subset sampling configuration (USE_HALF_DATASET=True, DATASET_SAMPLE_FRACTION=0.5), preprocessing pipeline, and evaluation scripts, will be made available via a public repository upon acceptance.

## Abbreviations

The following abbreviations are used in this manuscript:

APJ	Accuracy-per-Joule
AUC	Area Under Curve
cGAN	Conditional Generative Adversarial Network
CNN	Convolutional Neural Network
DDoS	Distributed Denial of Service
DoS	Denial of Service
EPS	Energy-per-Sample
F1PJ	F1-per-Joule
GAN	Generative Adversarial Network
GPU	Graphics Processing Unit
IDS	Intrusion Detection System
IoT	Internet of Things
LSTM	Long Short-Term Memory
MSE	Mean Squared Error
PCA	Principal Component Analysis
PGAN	Progressive Generative Adversarial Network
RNN	Recurrent Neural Network
ROC	Receiver Operating Characteristic
SMOTE	Synthetic Minority Over-sampling Technique
TCN	Temporal Convolutional Network
WGAN-GP	Wasserstein GAN with Gradient Penalty
